# HSP90α lactylation orchestrates PGC1α and LRPGC1 nuclear translocation driving mitochondrial biogenesis

**DOI:** 10.1073/pnas.2528979123

**Published:** 2026-07-21

**Authors:** Gang Wu, Hongmin Li, Tong He, Min Chen, Xiaoyu Jiang, Mengli Wei, Lei Zhou, Chengyu Li, Jingli Tao, Zhaojun Liu, Ming Shen, Honglin Liu

**Affiliations:** ^a^ Laboratory of Genetic Dissection and Regulation of Economically Important Traits in Animals, College of Animal Science and Technology, Nanjing Agricultural University, Nanjing 210095, China; ^b^Xinyu Agricultural Science Research Centre, Xinyu 338000, China

**Keywords:** HSP90α lactylation, HSP90α phosphorylation, ULK1, CDK5, PGC1α/LRPGC1

## Abstract

Mitochondrial biogenesis is a fundamental process that ensures energy supply and supports steroidogenesis in ovarian cells. Lactate has recently been identified as a signaling metabolite that promotes mitochondrial biogenesis; however, the underlying regulatory mechanisms remain poorly defined. Here, we identify Heat Shock Protein 90 Alpha Family Class A Member 1 (HSP90α) lactylation as a key mediator that links glycolytic metabolism to mitochondrial function. Specifically, lactylation of HSP90α at K58 recruits ULK1, thereby enhancing phosphorylation at S39; lactylation at K616 prevents CDK5-mediated phosphorylation at S596. This dual regulation facilitates the nuclear translocation of peroxisome proliferator-activated receptor gamma coactivator 1-alpha (PGC1α) and its isoform LRPGC1, which activate NRF1/2-dependent transcription of mitochondrial biogenesis genes, such as *Tfb1m*, *Tfb2m*, and *Tfam*. Functionally, mitochondrial mass expansion both enhances cellular energy metabolism and promotes cholesterol import into mitochondria, thereby driving estrogen biosynthesis. Together, these findings reveal a lactate–HSP90α–PGC1α/LRPGC1 axis that metabolically couples glycolysis to mitochondrial biogenesis and reproductive hormone production, providing insights into the epigenetic regulation of follicular development.

## Significance

Mitochondrial biogenesis is fundamental to ovarian follicle growth, as mitochondria supply both the energy required for granulosa cell proliferation and the substrates necessary for estrogen biosynthesis. Lactate has emerged as a signaling metabolite in energy metabolism; however, the mechanisms by which it regulates mitochondrial function in reproductive cells remain poorly understood. This study identifies HSP90αlactylation as a metabolite-dependent posttranslational modification that orchestrates PGC1α/LRPGC1 nuclear translocation and drives mitochondrial biogenesis. Our findings link glycolytic activity to mitochondrial expansion and steroidogenic capacity, revealing a layer of metabolic control in follicular development and the potential for targeting lactylation-dependent HSP90α regulation in ovarian function and fertility modulation.

Mitochondria serve as the metabolic hubs of eukaryotic cells. In addition generating ATP through oxidative phosphorylation (1), mitochondria contribute to numerous cellular processes, including metabolic intermediate conversion (2), precise calcium homeostasis modulation (3), and programmed apoptosis inhibition (4). Eukaryotic cells have evolved a sophisticated bidirectional mitochondria-nucleus communication system that coordinates these complex functions. In anterograde regulation (5), the nucleus dynamically controls mitochondrial functions through nuclear-encoded mitochondrial protein expression (6), including fine-tuning mitochondrial membrane potential and respiratory chain activity and promoting mitochondrial biogenesis according to energy demands (7, 8). In retrograde responses (5), mitochondria transmit key information about their metabolic status and ROS levels to the nucleus (9), remodeling the nuclear gene expression profile through epigenetic modifications and transcription factor activation (10), thereby systemically adjusting cellular metabolic patterns and functional states. This integrated network is particularly important in tissues with high, dynamically changing steroidogenic demands, such as the ovary, where mitochondrial function directly shapes follicular development and steroid hormone biosynthesis, According to the classical two-cell/two-gonadotropin framework (11), the StAR-mediated transport of cholesterol into the mitochondrial inner membrane and its conversion by CYP11A1 in theca cells initiate androgen production, which granulosa cells subsequently convert to estradiol (12). Moreover, emerging evidence indicates that granulosa cells can translocate cholesterol into their own mitochondria and generate estrogen precursor, adding a new layer of complexity to this coordinated intercellular process.

The nucleocytoplasmic shuttling of peroxisome proliferator-activated receptor gamma coactivator 1-alpha (PGC1α) plays a pivotal role in the intricate mitochondrial biogenesis regulatory network (13). Once localized to the nucleus, PGC1α functions as a central hub that cooperates with transcription factors such as nuclear respiratory factor 1 (NRF1) and GA-binding protein-α (GABPα; also known as NRF2), driving the expression of genes essential for mitochondrial biogenesis (8). More recently, a novel isoform, LRPGC1, has been identified as an additional regulator that shares nuclear localization properties and contributes to the dynamic remodeling of mitochondrial networks (14). Despite these advances, the mechanisms governing the precise nuclear import of PGC1α and LRPGC1 remain largely undefined.

Heat Shock Protein 90 Alpha Family Class A Member 1 (HSP90α) is a highly conserved and ubiquitously expressed molecular chaperone that plays essential roles in cellular physiology (15). HSP90α is reported to interact with approximately 10% of the eukaryotic proteome, positioning it as a central hub within the protein homeostasis network (15). Through coordinated interactions with various cochaperones and client proteins, HSP90α regulates multiple cellular processes including protein folding, stabilization, and functional activation, exhibiting functional diversity across different tissues and organs (16). In addition to these canonical chaperone functions, HSP90α regulates gene expression, contributing to the maturation and nucleocytoplasmic trafficking of transcription factors (17). However, it remains unclear whether HSP90α influences the nuclear import of key metabolic regulators central to mitochondrial biogenesis such as PGC1α and LRPGC1.

Recent studies have revea led a tight coupling between glycolysis and mitochondrial function (18). Lactate, traditionally considered as a glycolytic end product, is now recognized as a metabolic substrate and signaling molecule that can be taken up by cells and enter mitochondria, where it influences electron transport activity and oxidative metabolism (18). Importantly, lactate has been implicated in mitochondrial biogenesis regulation, indicating that its role in metabolic reprogramming extends beyond energy shuttling (19). Concomitantly, protein lysine lactylation (Kla), a lactate-derived posttranslational modification, has been identified as a mechanism by which the cellular metabolic state directly alters protein function and gene expression (20). Motivated by our earlier detection of lactylation on HSP90α (21), we sought to determine whether this modification could modulate the nucleocytoplasmic trafficking of PGC1α and LRPGC1, thereby coupling glycolytic flux to mitochondrial biogenesis.

This study reveals a regulatory mechanism in which HSP90α forms a functional complex with PGC1α/LRPGC1 and facilitates their nuclear translocation, a process essential for lactate-induced mitochondrial biogenesis. At the molecular level, lactylation of HSP90α exerts dual control over this process: modification at K58 recruits serine/threonine-protein kinase (ULK1), promoting phosphorylation at S39; modification at K616 prevents cyclin-dependent kinase 5 (CDK5) recruitment and blocks phosphorylation at S596. Through this bidirectional regulation, PGC1α and LRPGC1 are efficiently imported into the nucleus, where they enhance NRF1/2 activity and drive the expression of key mitochondrial biogenesis genes, including *Tfb1m*, *Tfb2m*, and *Tfam*. We further show that lactate is converted to Lactyl-CoA by ACSS2 and GTPSCS, and that it is subsequently transferred to HSP90α by CREBBP, establishing the metabolic basis of this modification. Functionally, activation of this lactate–HSP90α–PGC1α/LRPGC1 axis increases mitochondrial number and capacity and enhances cholesterol trafficking into mitochondria, thereby boosting estradiol biosynthesis. In vivo, these changes result in elevated circulating estrogen levels and a marked increase in antral follicle number, demonstrating the physiological relevance of this pathway in promoting follicular growth and ovarian function. Collectively, these findings reveal a direct molecular link between glycolytic metabolism, mitochondrial dynamics, and steroidogenic output, and highlight lactylation as a central regulatory layer that integrates energy metabolism with follicular development.

## Results

### Lactate Promotes Mitochondrial Biogenesis by Facilitating PGC1α and LRPGC1 Nuclear Translocation.

Effective coupling between glycolysis and mitochondrial metabolism is essential for supporting the energy demands of ovarian granulosa cell (GCs) proliferation and differentiation (22). However, how glycolysis-derived lactate precisely regulates mitochondrial function remains unclear. PGC1α is a master regulator of mitochondrial biogenesis; thus, we investigated whether lactate influences this process through PGC1α-dependent mechanisms.

We first examined the effects of sodium lactate on mitochondrial biogenesis. Lactate treatment significantly increased mitochondrial DNA copy number (*SI Appendix*, Fig. S1*A*), elevated protein levels of the mitochondrial marker TOM20 (*SI Appendix*, Fig. S1*B*), and enhanced MitoTracker staining, which reflects mitochondrial biogenesis rather than a mere alteration in membrane potential (*SI Appendix*, Fig. S1*C*). These pro-mitochondriogenic effects were largely abolished when lactate uptake was blocked with α-CHCA, an inhibitor of the lactate transporter MCT1 (*SI Appendix*, Fig. S1 *A*–*C*). Consistently, transmission electron microscopy (TEM) revealed an increase in the number of mitochondria after sodium lactate treatment (*SI Appendix*, Fig. S1*D*). Together, these findings demonstrate that lactate promotes mitochondrial biogenesis in an MCT1-dependent manner.

**Fig. 1.**
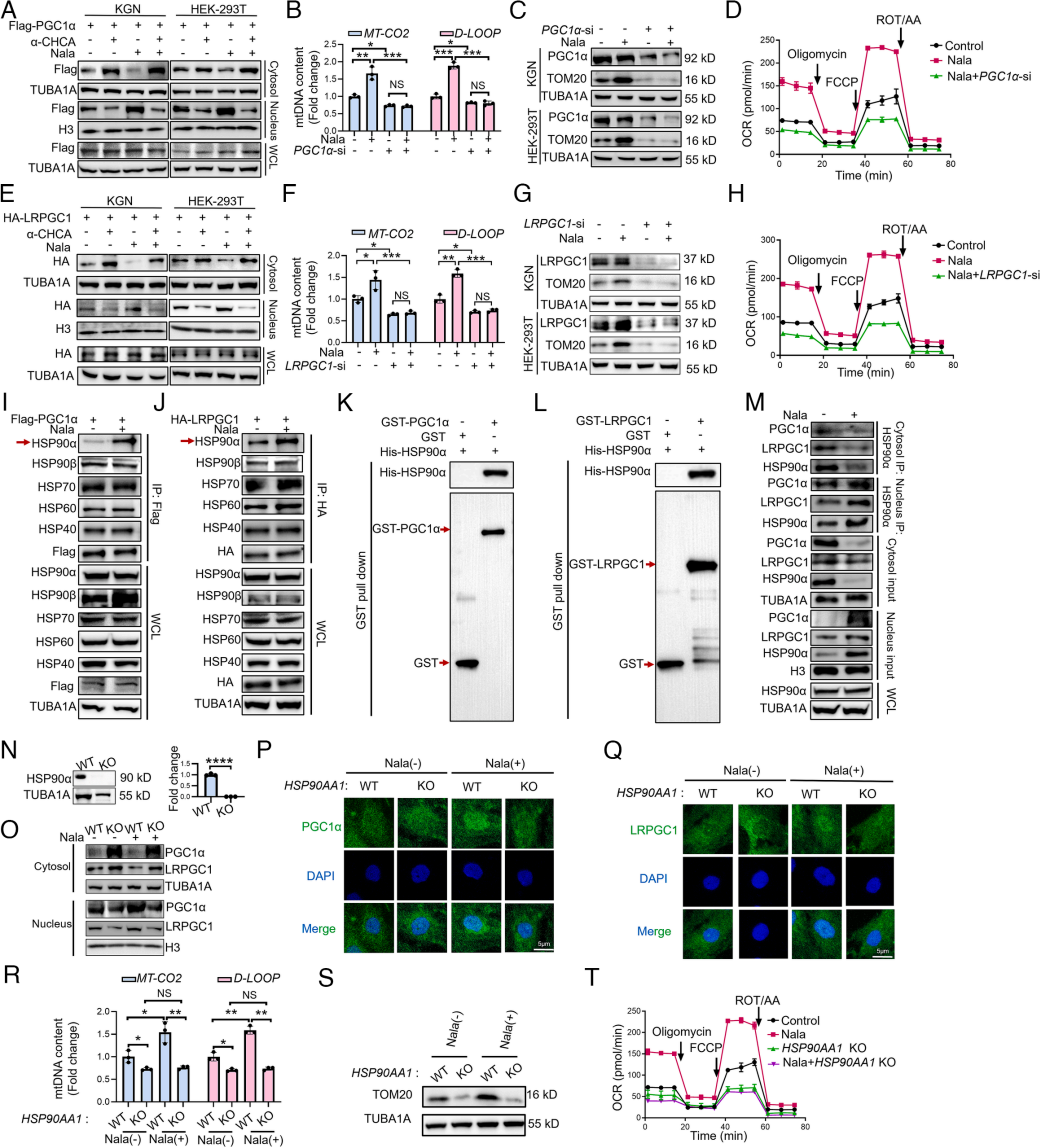
HSP90α modulates mitochondrial biogenesis through controlling the cyto-nuclear distribution of PGC1α/LRPGC1. (*A*) Nuclear and cytoplasmic fractionation analysis of Flag-PGC1α distribution in KGN and HEK-293 T cells pretreated with α-CHCA followed by sodium lactate. WCL: whole-cell lysate. (*B*–*D*) RT-qPCR analysis of mtDNA copy number (*MT-CO2*, *D-loop*) in KGN cells (*B*), western blot analysis of TOM20 in KGN and HEK-293 T cells (*C*), and OCR in KGN cells (*D*), after transfection with PGC1α siRNA followed by sodium lactate. (*E*) Nuclear and cytoplasmic fractionation analysis of HA-LRPGC1 distribution in KGN and HEK-293 T cells pretreated with α-CHCA followed by sodium lactate. (*F*–*H*) RT-qPCR of mtDNA copy number (*MT-CO2*, *D-loop*) in KGN cells (*F*), western blot of TOM20 in KGN and HEK-293 T cells (*G*), and OCR in KGN cells (*H*), after transfection with LRPGC1 siRNA followed by sodium lactate. (*I* and *J*) Coimmunoprecipitation analysis of Flag-PGC1α (*I*) and HA-LRPGC1 (*J*) binding to heat shock proteins in KGN cells with or without sodium lactate. (*K* and *L*) GST pull-down analysis of His–HSP90α binding to GST-PGC1α (*K*) and GST-LRPGC1 (*L*) using purified recombinant or prokaryotic proteins. (*M*) Coimmunoprecipitation analysis of HSP90α binding to PGC1α/LRPGC1 in nuclear/cytoplasmic fractions of KGN cells with or without sodium lactate. (*N*) HSP90α expression levels in wild-type (WT) vs. *HSP90AA1* KO KGN cells. (*O*) Nuclear and cytoplasmic fractionation analysis of PGC1α and LRPGC1 distribution in *HSP90AA1* KO KGN cells with or without sodium lactate. (*P*–*T*) Immunofluorescence of PGC1α (*P*) and LRPGC1 (*Q*) distribution (nuclei: DAPI; Scale bar, 5 μm), mtDNA copy number by RT-qPCR (*R*), TOM20 by western blot (*S*), and OCR (*T*) in WT and *HSP90AA1* KO KGN cells with or without sodium lactate. Nala: sodium lactate. The data were presented as mean ± SD. Differences between groups were assessed using ANOVA. *P < 0.05; **P < 0.01; ***P < 0.001; ****P < 0.0001. NS indicates no difference.

We next investigated whether PGC1α mediates this effect. Sodium lactate markedly enhanced the nuclear translocation of PGC1α (Fig. 1*A* and *SI Appendix*, Fig. S1 *E* and *F*), whereas this response was suppressed by α-CHCA (Fig. 1*A* and *SI Appendix*, Fig. S1 *E* and *F*). *PGC1α* knockdown via siRNA significantly impaired lactate-induced mitochondrial biogenesis, as indicated by reduced mtDNA copy number (Fig. 1*B*), diminished TOM20 expression (Fig. 1*C* and *SI Appendix*, Fig. S1*G*), and decreased MitoTracker fluorescence (*SI Appendix*, Fig. S1*H*). Furthermore, lactate treatment increased the oxygen consumption rate (OCR), reflecting enhanced mitochondrial respiration, whereas PGC1α knockdown abolished this effect (Fig. 1*D*). These results establish that lactate promotes mitochondrial biogenesis by driving PGC1α nuclear translocation.

The lactate-responsive PGC1α isoform, LRPGC1 comprises the N-terminal 269 amino acids of PGC1α (14); thus, we evaluated its role in the aforementioned process. Sodium lactate induced LRPGC1 nuclear accumulation, similar to that observed with PGC1α (Fig. 1*E* and *SI Appendix*, Fig. S2 *A* and *B*) blocking lactate uptake with α-CHCA or the alternative MCT1 inhibitor AZD-3965 inhibited this effect (Fig. 1*E* and *SI Appendix*, Fig. S2 *A*–*C*). Under hypoxic conditions, which enhance glycolytic metabolism, LRPGC1 also accumulated in the nucleus, and this effect was reversed by suppressing lactate production via LDHA/B knockdown (*SI Appendix*, Fig. S2*D*). Functionally, depletion of *LRPGC1* by siRNA abolished lactate-induced mitochondrial biogenesis, as reflected by reduced mtDNA copy number (Fig. 1*F*), decreased TOM20 expression (Fig. 1*G* and *SI Appendix*, Fig. S2*E*), diminished MitoTracker staining (*SI Appendix*, Fig. S2*F*), and loss of the lactate-induced OCR increase (Fig. 1*H*).

Next, we knocked down PGC1α, LRPGC1, or both simultaneously to further assess their effects on TOM20 protein expression. Knockdown of either PGC1α or LRPGC1 suppressed TOM20 protein expression (*SI Appendix*, Fig. S2*G*). Double knockdown did not yield a further reduction compared with individual knockdown (*SI Appendix*, Fig. S2*G*), suggesting that PGC1α and LRPGC1 cooperatively regulate mitochondrial biogenesis. These findings establish that lactate drives the nuclear import of PGC1α and LRPGC1, thereby activating mitochondrial biogenesis.

### HSP90α Is Required for Lactate-Induced PGC1α or LRPGC1 Nuclear Translocation.

Lactate has recently been recognized as a direct substrate for protein lactylation, which regulates diverse cellular processes (20). Thus, we investigated whether PGC1α/LRPGC1 nuclear translocation is modulated by lactylation. However, coimmunoprecipitation analyses revealed no detectable lactylation on either PGC1α or LRPGC1 following sodium lactate treatment (*SI Appendix*, Fig. S3 *A* and *B*), prompting us to explore alternative regulatory mechanisms. Given the pivotal roles of heat shock proteins (HSPs) in autophagy (23), protein trafficking (24), apoptosis (25), and mitochondrial biogenesis (26), coupled with reported evidence that HSP family proteins are essential for stabilizing the nuclear localization of PGC1α (27), we hypothesized that HSP family members might mediate the nuclear import of PGC1α/LRPGC1. Coimmunoprecipitation assays demonstrated significantly enhanced binding between both PGC1α/LRPGC1 and HSP90α upon sodium lactate stimulation, whereas interactions with HSP90β, HSP70, HSP60, or HSP40 remained unchanged (Fig. 1 *I* and *J* and *SI Appendix*, Fig. S3*C*). This lactate-enhanced interaction was abolished by α-CHCA (*SI Appendix*, Fig. S3 *D* and *E*). To demonstrate that PGC1α and LRPGC1 each directly bind to HSP90α, we expressed and purified GST-tagged PGC1α and LRPGC1, along with His-tagged HSP90α, using a prokaryotic expression system (*SI Appendix*, Fig. S3 *F* and *G*), and subsequently performed GST pull-down assays. Both PGC1α and LRPGC1 were found to directly bind to HSP90α (Fig. 1 *K* and *L*). Next, to explore the role of HSP90α in lactate-induced nuclear translocation of PGC1α and LRPGC1, we conducted coimmunoprecipitation assays using nuclear and cytoplasmic fractionation following sodium lactate treatment. Sodium lactate treatment induced robust nuclear accumulation of HSP90α, PGC1α, and LRPGC1 (Fig. 1*M* and *SI Appendix*, Fig. S3*H*), while concurrently enhancing the physical interaction between HSP90α and both PGC1α and LRPGC1 specifically within the nuclear compartment (Fig. 1*M* and *SI Appendix*, Fig. S3*H*).

Given that HSP90α is a key regulator of cellular stress responses, we further examined its nuclear localization under heat and LPS-induced stress conditions. Neither heat stress nor LPS treatment alone altered lactate production or HSP90α nuclear translocation (*SI Appendix*, Fig. S4*A*). However, upon additional lactate treatment under these stress conditions, intracellular lactate levels were significantly increased, accompanied by enhanced HSP90α nuclear translocation (*SI Appendix*, Fig. S4*A*). These findings suggest that under stress conditions, HSP90α primarily functions in the cytoplasm, whereas lactate induces its nuclear translocation, which may be involved in gene expression and mitochondrial biogenesis regulation.

We next used the inhibitor 17-AAG to suppress HSP90α activity. Coimmunoprecipitation assays revealed that 17-AAG treatment significantly attenuated the interaction between PGC1α and LRPGC1 and HSP90α (*SI* *Appendix*, Fig. S4 *B*–*D*). Nuclear and cytoplasmic fractionation experiment further demonstrated that inhibiting HSP90α activity markedly impaired the translocation of PGC1α and LRPGC1 from the cytoplasm to the nucleus (*SI Appendix*, Fig. S4*E*). Additionally, immunofluorescence analyses confirmed the significant suppression of PGC1α and LRPGC1 nuclear localization in 17-AAG-treated cells even in the presence of sodium lactate stimulation (*SI Appendix*, Fig. S4 *F* and *G*). Notably, inhibition of HSP90α activity also impaired lactate-induced mitochondrial biogenesis, as evidenced by reduced mitochondrial DNA copy number (*SI Appendix*, Fig. S4*H*), suppressed expression of the mitochondrial marker protein TOM20 (*SI Appendix*, Fig. S4*I*), and diminished MitoTracker fluorescence intensity (*SI Appendix*, Fig. S4*J*).

To mitigate the potential for off-target and nonspecific effects associated with 17-AAG, we employed multiple genetic approaches to further validate the functional role of HSP90α. Initially, we used targeted siRNA to effectively knock down HSP90α expression (*SI Appendix*, Fig. S5 *A* and *B*), which significantly inhibited sodium lactate-induced nuclear translocation of PGC1α and LRPGC1 (*SI Appendix*, Fig. S5 *C*–*F*). To more rigorously exclude potential off-target effects of siRNA, we further generated *HSP90AA1* knockout (KO) KGN cell lines using CRISPR-Cas9 technology (Fig. 1*N*). In *HSP90AA1* KO KGN cells, nuclear and cytoplasmic fractionation experiments demonstrated that sodium lactate-induced nuclear translocation of PGC1α and LRPGC1 was completely blocked (Fig. 1*O* and *SI Appendix*, Fig. S5*G*), a finding further confirmed by immunofluorescence assays (Fig. 1 *P* and *Q* and *SI Appendix*, Fig. S5 *H* and *I*). Consistently, *HSP90AA1* KO abolished lactate-induced mitochondrial biogenesis, supported by decreased mitochondrial DNA copy number (Fig. 1*R*), reduced TOM20 expression (Fig. 1*S* and *SI Appendix*, Fig. S5*J*), and attenuated MitoTracker fluorescence signal (*SI Appendix*, Fig. S5*K*). Similarly, *HSP90AA1* KO also abrogated the sodium lactate-induced increase in OCR (Fig. 1*T*).

To further investigate whether HSP90α functions in a manner dependent on HSP90β, we knocked down HSP90α, HSP90β, or both, and then subjected cells to lactate treatment. We first examined the knockdown efficiency of HSP90β (*SI Appendix*, Fig. S5 *L* and *M*). Knockdown of HSP90α significantly reduced lactate-induced TOM20 protein expression, whereas knockdown of HSP90β had no notable effect (*SI Appendix*, Fig. S5 *N* and *O*). Moreover, simultaneous knockdown of both HSP90α and HSP90β did not further alter TOM20 expression compared with HSP90α knockdown alone (*SI Appendix*, Fig. S5 *N* and *O*). Thus, HSP90α functions independently of HSP90β. Overall, these results demonstrate that HSP90α directly interacts with PGC1α and LRPGC1 and is required for their lactate-induced nuclear translocation and subsequent mitochondrial biogenesis.

### HSP90α Is Lactylated by CBP Acetyltransferase.

**Fig. 2.**
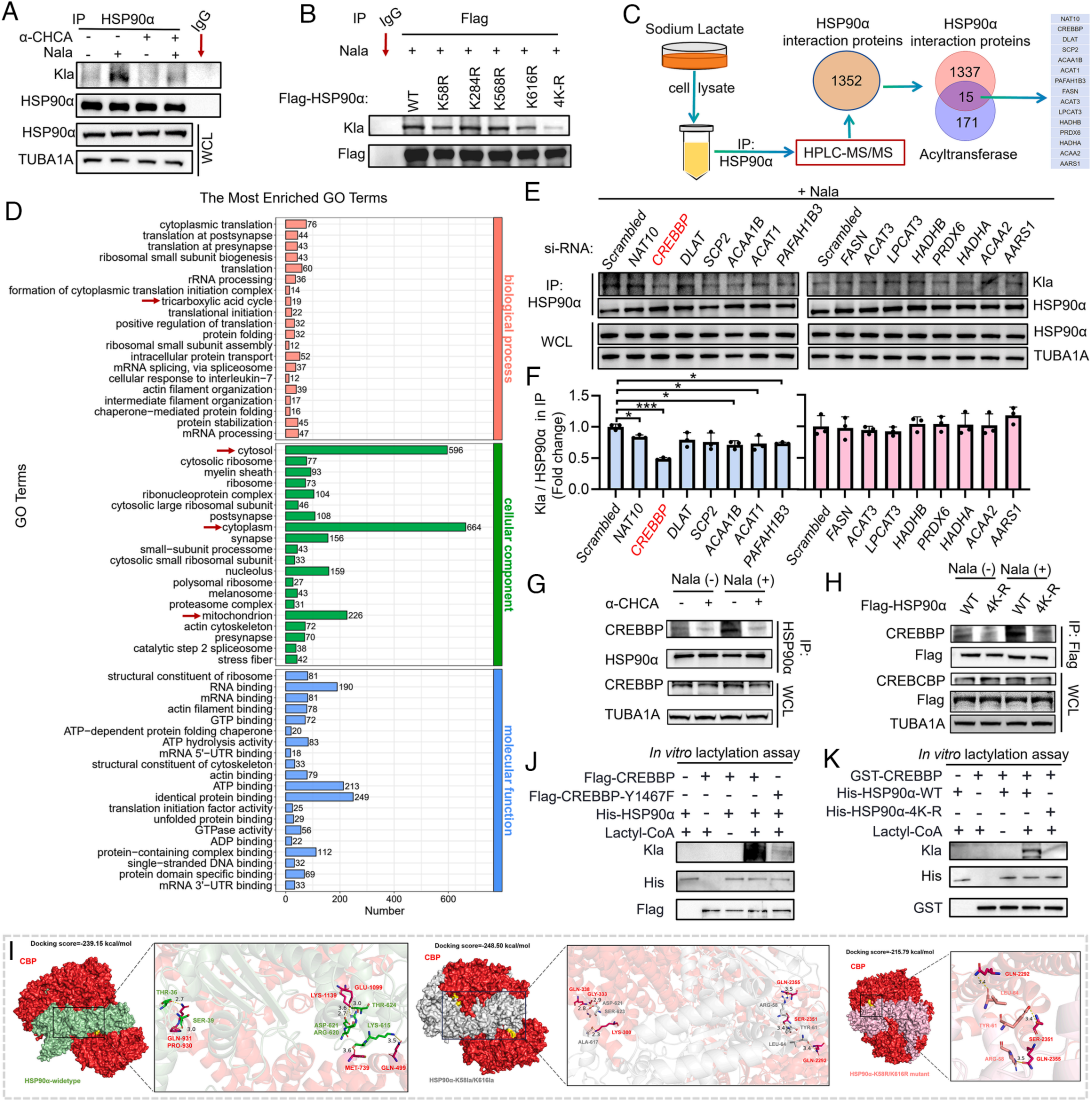
Identification of CBP as the lactyltransferase responsible for HSP90α lysine 58 and lysine 616 lactylation. (*A*) Coimmunoprecipitation analysis of HSP90α lactylation in KGN cells pretreated with α-CHCA followed by sodium lactate. (*B*) Coimmunoprecipitation analysis of HSP90α lactylation in KGN cells expressing WT or mutant (K58R, K284R, K568R, K616R, 4 K–R) Flag-HSP90α, followed by sodium lactate treatment. (*C*) Schematic of IP–HPLC–MS/MS workflow for identifying HSP90α-interacting lactyltransferases. (*D*) GO analysis of HSP90α-interacting proteins. (*E* and *F*) Coimmunoprecipitation of HSP90α lactylation (*E*) and its quantification (*F*) in KGN cells after knockdown of 15 HSP90α-binding acyltransferases with siRNAs and sodium lactate treatment. (*G*) Coimmunoprecipitation analysis of HSP90α–CREBBP interaction in KGN cells pretreated with α-CHCA followed by sodium lactate. (*H*) Coimmunoprecipitation analysis of Flag-HSP90α–CREBBP interaction in KGN cells expressing WT or 4 K–R Flag-HSP90α, with or without sodium lactate. (*I*) Molecular docking analysis of CREBBP binding to WT, K58/K616 lactylation-mimetic (K58la/K616la), and K58/K616 arginine mutant (K58R/K616R) HSP90α models. (*J*) In vitro lactylation assay of purified HSP90α with Lactyl-CoA using Flag–CREBBP immobilized on magnetic beads. (*K*) In vitro lactylation assay of His–HSP90α by GST-CREBBP HAT domain in the presence of Lactyl-CoA. Nala: sodium lactate. The data were presented as mean ± SD. Differences between groups were assessed using ANOVA. *P < 0.05; **P < 0.01; ***P < 0.001; ****P < 0.0001. NS indicates no difference.

We next investigated whether HSP90α undergoes lactylation. Coimmunoprecipitation in KGN cells revealed that sodium lactate treatment significantly increased HSP90α lactylation, whereas blocking lactate uptake effectively suppressed this modification (Fig. 2*A*).

Lactyl-CoA has been proposed as the substrate for protein lactylation (28); thus, we assessed the involvement of its synthesis. Sodium lactate treatment significantly increased Lactyl-CoA levels and enhanced HSP90α lactylation (*SI Appendix*, Fig. S6 *A*–*C*). However, these effects were markedly inhibited by knockdown of the lactyl-CoA synthetase *ACSS2* (29) or *SUCLG1* (a catalytic subunit of the GTP synthase complex, GTPSCS) (30) (*SI Appendix*, Fig. S6 *A*–*C*), indicating that lactate promotes HSP90α lactylation through the Lactyl-CoA synthesis pathway. Mass spectrometry identified four lysine residues (K58, K284, K568, and K616) as lactylation sites on HSP90α (*SI Appendix*, Fig. S6*D*). These sites and their flanking amino acid sequences are highly conserved across diverse species (*SI Appendix*, Fig. S6*E*). To investigate the functional roles of these sites, we constructed K58R, K284R, K568R, K616R, and a quadruple mutant (4 K–R) to mimic the delactylated state. Subsequently, we transfected KGN cells with Flag-tagged WT and mutant constructs and assessed lactylation levels via coimmunoprecipitation. In sodium lactate-treated KGN cells, K58R and K616R mutations significantly inhibited HSP90α lactylation, and the 4 K–R mutant almost completely abolished it (Fig. 2*B*). K284R and K568R mutations showed no significant effect (Fig. 2*B*), indicating that K58 and K616 are the primary lactylation sites on HSP90α.

To identify the lactyltransferase responsible for HSP90α lactylation, we immunoprecipitated HSP90α and conducted an LC–MS/MS analysis in sodium lactate-treated KGN cells, identifying 1,352 HSP90α-interacting proteins (Fig. 2*C* and Dataset S1). Gene Ontology (GO) enrichment analysis showed significant enrichment of these proteins in pathways related to protein transport and mitochondrial biogenesis (Fig. 2*D* and *SI Appendix*, Fig. S7 *A*–*C*). Kyoto Encyclopedia of Genes and Genomes (KEGG) analysis further demonstrated their close association with the tricarboxylic acid cycle and glycolysis (*SI Appendix*, Fig. S7*D*), suggesting that HSP90α plays an important role in regulating mitochondrial function. We then cross-referenced against known acyltransferases, revealing 15 candidates potentially involved in HSP90α lactylation from the interactome (Fig. 2*C*).

To determine the key regulatory enzyme, we designed specific siRNAs targeting these 15 candidate acyltransferases and confirmed their knockdown efficiencies (*SI Appendix*, Fig. S8*A*). Immunoprecipitation coupled with western blotting. revealed that knockdown of CREB-binding protein (CREBBP, also referred to as CBP), previously reported as a “writer” enzyme for protein lactylation, was uniquely required; compared with other enzymes, its knockdown markedly reduced HSP90α lactylation (Fig. 2 *E* and *F*). Further experiments demonstrated that sodium lactate treatment markedly enhanced CREBBP–HSP90α binding (Fig. 2*G* and *SI Appendix*, Fig. S8*B*), while the lactate uptake inhibitor α-CHCA blocked this interaction (Fig. 2*G* and *SI Appendix*, Fig. S8*B*). In KGN cells expressing either WT or 4 K–R mutant HSP90α, lactate-induced binding to CREBBP was lost in the 4 K–R mutant (Fig. 2*H* and *SI Appendix*, Fig. S8*C*).

To further investigate the role of lactylation sites, we subjected the K58 and K616 residues of HSP90α to two types of modifications: simultaneous conjugation with lactoyl groups (mimicking lactylation) or simultaneous mutation of both residues to arginine (R). Molecular docking was then performed to assess the binding between HSP90α and CREBBP. Compared with the WT, the introduction of lactoyl groups at K58 and K616 enhanced binding energy (Fig. 2*I*), whereas simultaneous mutation of K58 and K616 to arginine (R) reduced it (Fig. 2*I*), providing direct evidence that HSP90α lactylation facilitates its interaction with CREBBP.

Next, to determine whether CREBBP functions as a lactyltransferase for HSP90α, we generated a WT Flag–CREBBP construct and an active-site mutant (Y1467F). Overexpressed in HEK-293 T cells and immobilized on Flag beads, WT CREBBP, but not mutant CREBBP promoted HSP90α lactylation in vitro (Fig. 2*J*). To exclude contributions from CREBBP-interacting proteins, we purified HAT domain of CREBBP, from *Escherichia coli*. The HAT domain of CREBBP was sufficient to promote HSP90α lactylation, whereas a 4 K–R mutant of HSP90α did not (Fig. 2*K*). Together, these findings indicate that CREBBP acts as a “writer” enzyme for HSP90α lactylation.

### HSP90α Lactylation Regulates the Nuclear Import of PGC-1α or LRPGC1, Promoting Mitochondrial Biogenesis.

**Fig. 3.**
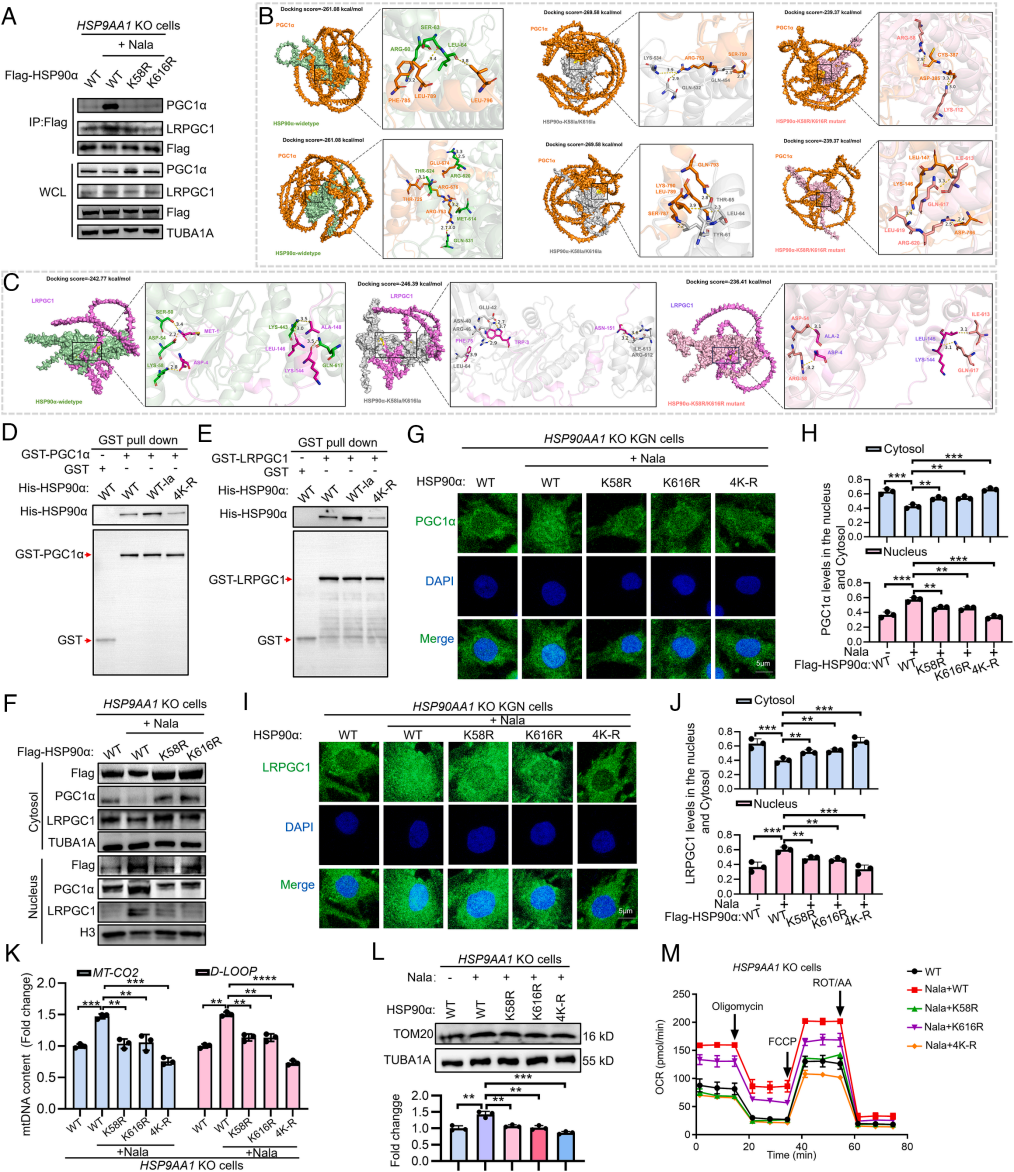
Lactate-induced lactylation of HSP90α regulates mitochondrial biogenesis. (*A*) Coimmunoprecipitation analysis of HSP90α binding to PGC1α/LRPGC1 in KGN cells expressing WT, K58R, or K616R, followed by sodium lactate treatment. (*B* and *C*) Molecular docking of WT, K58la/K616la, and K58R/K616R HSP90α with PGC1α (*B*) or LRPGC1 (*C*). (*D* and *E*) GST pull-down analysis of GST-PGC1α (*D*) and GST-LRPGC1 (*E*) with WT HSP90α, lactylated HSP90α (WT-la), or 4 K-R HSP90α. (*F*) Nuclear and cytoplasmic fractionation analysis of PGC1α, LRPGC1, and HSP90α distribution in KGN cells expressing WT, K58R, or K616R, followed by sodium lactate treatment. (*G*–*M*) Immunofluorescence analysis of PGC1α (*G*) and LRPGC1 (*I*) subcellular localization, quantitative analysis of fluorescence intensity (*H* and *J*), RT-qPCR of mtDNA copy number (*K*), western blot of TOM20 (*L*), and OCR (*M*) in KGN cells expressing WT, K58R, K616R, or 4 K-R, followed by sodium lactate treatment. Nala: sodium lactate. The data were presented as mean ± SD. Differences between groups were assessed using ANOVA. *P < 0.05; **P < 0.01; ***P < 0.001; ****P < 0.0001. NS indicates no difference.

To investigate how lactylation affects HSP90α function, we reexpressed WT or mutant HSP90α in *HSP90AA1* KO KGN cells. Coimmunoprecipitation showed that both K58R and K616R mutations impaired the interaction between HSP90α and PGC1α/LRPGC1 (Fig. 3*A* and *SI Appendix*, Fig. S8*D*). Molecular docking analysis further revealed that the addition of lactoyl groups to K58 and K616 to mimic lactylation increased the binding energy of both HSP90α-PGC1α and HSP90α-LRPGC1 (Fig. 3 *B* and *C*). In contrast, the K58R/K616R mutations reduced the binding affinity for both interactions (Fig. 3 *B* and *C*). To further demonstrate that HSP90α lactylation enhances the binding of HSP90α to PGC1α or LRPGC1, we purified prokaryotically expressed His-tagged HSP90α and His-tagged 4 K–R mutant HSP90α and subjected the His-tagged HSP90 to an in vitro lactylation reaction. Meanwhile, we purified GST-tagged LRPGC1 and PGC1α, followed by GST pull-down assays. The PGC1α and LRPGC1 exhibited enhanced binding to lactylated HSP90α (WT-la), whereas the 4 K-R mutation reduced their binding to HSP90 (Fig. 3 *D* and *E*).

We next examined the regulatory role of HSP90α lactylation in the nuclear localization of PGC1α or LRPGC1. In *HSP90AA1* KO KGN cells, reexpression of WT HSP90α restored lactate-induced nuclear translocation of PGC1α and LRPGC1, whereas K58R and K616R mutations impaired this process, and the 4 K–R mutant resulted in the strongest inhibition (Fig. 3 *F*–*J* and *SI Appendix*, Fig. S8*E*). Functionally, these mutations also suppressed mitochondrial biogenesis, as indicated by reduced mtDNA copy number (Fig. 3*K*), decreased TOM20 expression (Fig. 3*L*), diminished MitoTracker staining (*SI Appendix*, Fig. S9*A*), and loss of the lactate-stimulated OCR increase (Fig. 3*M*).

Next, we used siRNA to knock down CREBBP and examined the nuclear translocation of PGC1α and LRPGC1. Immunofluorescence revealed that CREBBP knockdown significantly impaired nuclear accumulation of both cofactors in response to lactate (*SI Appendix*, Fig. S9 *B* and *C*). Consistently, CREBBP depletion attenuated mitochondrial biogenesis, as evidenced by reduced mtDNA copy number (*SI Appendix*, Fig. S9*D*), lower TOM20 expression (*SI Appendix*, Fig. S9*E*), and decreased MitoTracker fluorescence intensity (*SI Appendix*, Fig. S9*F*). OCR analysis further confirmed that CREBBP knockdown suppressed the lactate-induced enhancement of mitochondrial respiration (*SI Appendix*, Fig. S9*G*). These results identify CREBBP as the lactyltransferase for HSP90α and demonstrate its requirement for PGC1α and LRPGC1 nuclear translocation and mitochondrial biogenesis.

### HSP90α Lactylation Promotes Mitochondrial Biogenesis by Opposing the Regulation of S39 and S596 Phosphorylation.

Previous studies have revealed two critical phosphorylation sites in HSP90AA1—serine 39 (S39) and serine 596 (S596), that play essential roles in regulating its function (17, 31). Specifically, ULK1 kinase-mediated phosphorylation at S39 significantly activates autophagy (31), while CDK5-catalyzed phosphorylation at S596 inhibits autophagy by suppressing TFEB nuclear translocation (17). These findings indicate that phosphorylation at S39 and S596 exerts opposing effects on HSP90α function. Sequence alignment analysis further revealed that both sites and their flanking regions are highly conserved across species (*SI Appendix*, Fig. S10*A*).

**Fig. 4.**
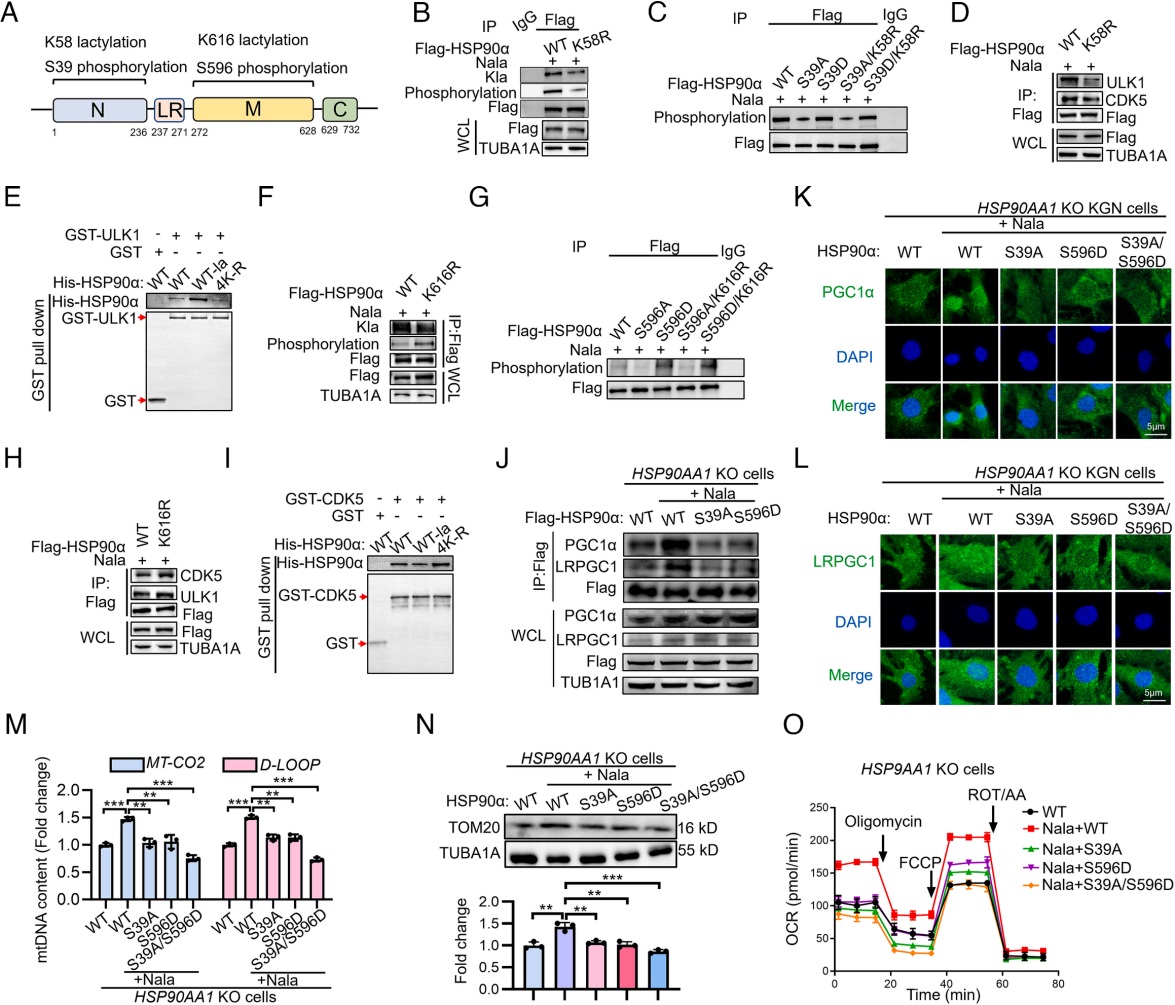
Lactate-induced HSP90α lactylation promotes mitochondrial biogenesis by regulating its phosphorylation. (*A*) Structural domain analysis of HSP90α modification sites: K58 lactylation, S39 phosphorylation, K616 lactylation, and S596 phosphorylation. N: NH_2_-terminus, LR: charged linker region, M: middle domain, C: COOH-terminus. (*B*) Coimmunoprecipitation analysis of lactylation and phosphorylation of Flag-HSP90α in KGN cells expressing WT or K58R, treated with sodium lactate. (*C*) Coimmunoprecipitation analysis of Flag-HSP90α phosphorylation in KGN cells expressing WT, S39A, S39D, S39A/K58R, or S39D/K58R, treated with sodium lactate. (*D*) Coimmunoprecipitation analysis of ULK1 and CDK5 binding to Flag-HSP90α in KGN cells expressing WT or K58R, treated with sodium lactate. (*E*) GST pull-down analysis of GST–ULK1 binding to WT, lactylated (WT-la), or 4 K–R HSP90α. (*F*) Coimmunoprecipitation analysis of lactylation and phosphorylation of Flag-HSP90α in KGN cells expressing WT or K616R, treated with sodium lactate. (*G*) Coimmunoprecipitation analysis of Flag-HSP90α phosphorylation in KGN cells expressing WT, S596A, S596D, S596A/K616R, or S596D/K616R, treated with sodium lactate. (*H*) Coimmunoprecipitation analysis of ULK1 and CDK5 binding to Flag-HSP90α in KGN cells expressing WT or K616R, treated with sodium lactate. (*I*) GST pull-down analysis of GST–CDK5 binding to WT, lactylated (WT-la), or 4 K–R HSP90α. (*J*) Coimmunoprecipitation analysis of HSP90α binding to PGC1α/LRPGC1 in lactate-treated *HSP90AA1* KO KGN cells expressing WT, S39A, or S596D. (*K*–*O*) Immunofluorescence of PGC1α (*K*) and LRPGC1 (*L*) localization, mtDNA copy number by RT-qPCR (*M*), TOM20 by western blot (*N*), and OCR (*O*) in lactate-treated *HSP90AA1* KO KGN cells expressing WT, S39A, S596D, or S39A/S596D HSP90α. The data were presented as mean ± SD. Differences between groups were assessed using ANOVA. *P < 0.05; **P < 0.01; ***P < 0.001; ****P < 0.0001. NS indicates no difference.

To examine whether lactate regulates mitochondrial biogenesis by modulating HSP90α phosphorylation, we first assessed the effect of sodium lactate treatment and α-CHCA inhibition. Neither sodium lactate treatment nor inhibition of lactate uptake altered the global phosphorylation level of HSP90α (*SI Appendix*, Fig. S10 *B* and *C*). The structural analysis revealed an intriguing spatial distribution pattern: the K58 lactylation site colocalizes with the S39 phosphorylation site in the N-terminal domain, whereas the K616 lactylation site colocalizes with the S596 phosphorylation site in the middle domain (Fig. 4*A* and *SI Appendix*, Fig. S10*A*). This domain-specific colocalization suggests that K58 lactylation may selectively regulate S39 phosphorylation, whereas K616 lactylation may specifically modulate S596 phosphorylation.

To test this hypothesis, we transfected KGN cells with Flag-tagged WT HSP90α or the K58R mutant plasmids, then treated with sodium lactate, and performed coimmunoprecipitation assays. The K58R mutation significantly reduced both lactylation and phosphorylation levels of Flag-HSP90α, indicating that K58 lactylation may positively regulate HSP90α phosphorylation (Fig. 4*B* and *SI Appendix*, Fig. S10*D*). To determine whether this effect was specific to S39, we generated S39A (dephosphorylation mimic), S39D (phosphorylation mimic), and their double mutants with K58R. Coimmunoprecipitation assays showed no additional reduction in phosphorylation in the S39A/K58R double mutant compared with S39A alone, and no difference between S39D and S39D/K58R (Fig. 4*C* and *SI Appendix*, Fig. S10*E*). Furthermore, the K58R mutation specifically impaired the interaction between Flag-HSP90α and ULK1 but had no effect on CDK5 binding (Fig. 4*D* and *SI Appendix*, Fig. S10*F*). Pull-down assays showed that ULK1 bound more strongly to lactylated HSP90α, whereas the K58R mutation inhibited the binding between ULK1 and HSP90α (Fig. 4*E* and *SI Appendix*, Fig. S10*G*). Molecular binding analysis showed that introducing a lactyl group at residue K58 enhanced the binding between HSP90α and ULK1 (*SI Appendix*, Fig. S10*H*). In contrast mutation at this site reduced the binding energy, indicating decreased affinity (*SI Appendix*, Fig. S10*H*). These results demonstrate that the K58 site positively regulates S39 phosphorylation.

We next examined the role of HSP90α K616 lactylation on its phosphorylation. In *HSP90AA1* KO cells, reexpression of the K616R mutant enhanced overall HSP90α phosphorylation compared with that observed in the WT (Fig. 4*F* and *SI Appendix*, Fig. S10*I*). Given that phosphorylation at the S596 site of HSP90α exerts a negative regulatory effect on its function, we hypothesized that this phosphorylation might be inhibited by lactylation at the K616 site. To test this hypothesis, we constructed the following four HSP90α mutant plasmids and subjected them to coimmunoprecipitation assays: S596A (a phosphodeficient mutant), S596D (a phosphomimetic mutant), S596A/K616R (a phosphodeficient and delactylation double mutant), and S596D/K616R (a phosphomimetic and delactylation double mutant). Compared with the S596A single mutant, the S596A/K616R double mutant did not further alter the phosphorylation level of HSP90α (Fig. 4*G* and *SI Appendix*, Fig. S10*J*). Similarly, the S596D/K616R double mutant also failed to produce any additional change in phosphorylation compared with the S596D single mutant (Fig. 4*G* and *SI Appendix*, Fig. S10*J*). Notably, the K616R mutation enhanced the interaction between Flag-HSP90α and CDK5 without affecting ULK1 binding (Fig. 4*H* and *SI Appendix*, Fig. S10*K*). In vitro pull-down assays further confirmed that the K616R mutation strengthened the interaction between His–HSP90α and GST–CDK5 (Fig. 4*I* and *SI Appendix*, Fig. S10*G*). Molecular docking showed that after K616 mutation, the binding energy between HSP90α and CDK5 increased, indicating enhanced affinity, whereas the introduction of a lactyl group at K616 decreased the binding energy, indicating reduced affinity (*SI Appendix*, Fig. S10*L*). Collectively, these data demonstrate a dual regulatory mechanism: lactate-induced K58 lactylation facilitates ULK1 recruitment and subsequent S39 phosphorylation, whereas K616 lactylation impedes CDK5 binding, attenuating S596 phosphorylation.

### HSP90α Phosphorylation Facilitates Lactate-Induced Mitochondrial Biogenesis.

To assess the role of HSP90α phosphorylation in lactate-induced mitochondrial biogenesis, we transfected *HSP90AA1*-knockout KGN cells with Flag-tagged WT HSP90α or its phosphomimetic/dephosphomimetic mutants (S39A and S596D), followed by sodium lactate treatment. Coimmunoprecipitation assays revealed that both the S39A and S596D mutations impaired the interaction between HSP90α and PGC1α or LRPGC1 (Fig. 4*J* and *SI Appendix*, Fig. S11*A*), indicating that both phosphorylation sites cooperatively regulate the formation of HSP90α–PGC1α and HSP90α–LRPGC1 complexes. Nuclear–cytoplasmic fractionation experiments demonstrated that either the S39A or S596D mutation inhibited the sodium lactate-induced nuclear translocation of HSP90α, PGC1α, and LRPGC1 (*SI Appendix*, Fig. S11 *B* and *C*). Immunofluorescence staining further demonstrated that both single mutations prevented the nuclear import of PGC1α or LRPGC1 even under sodium lactate stimulation (Fig. 4 *K* and *L* and *SI Appendix*, Fig. S11 *D* and *E*). In contrast, the double mutation S39A/S596D exhibited a more pronounced inhibitory effect (Fig. 4 *K* and *L* and *SI Appendix*, Fig. S11 *D* and *E*). Through mitochondrial DNA copy number analysis (Fig. 4*M*), TOM20 protein level detection (Fig. 4*N*), MitoTracker fluorescence assays (*SI Appendix*, Fig. S11*F*), and OCR functional assays (Fig. 4*O*), we demonstrated that both S39A and S596D mutations significantly suppressed sodium lactate-induced mitochondrial biogenesis, with the double mutant showing a stronger inhibitory effect. These results suggest that phosphorylation at both S39 and S596 sites of HSP90α is essential for regulating mitochondrial biogenesis.

Nuclear PGC1α exerts its function by binding to NRF1/2 (8); thus, we next examined the interaction between PGC1α and NRF1/2. Sodium lactate treatment enhanced the binding of PGC1α or LRPGC1 to NRF1/2 (*SI Appendix*, Fig. S12 *A*–*F*), whereas K58R and K616R mutations inhibited this lactate-induced interaction (*SI Appendix*, Fig. S12 *A* and *B*). Similarly, the S39A and S596D mutations also impaired the sodium lactate-facilitated binding between PGC1α or LRPGC1 and NRF1/2 (*SI Appendix*, Fig. S12 *C* and *D*). Compared with the WT, neither the single mutations (S39D or K616A) nor the double mutation (S39D/K616A) further enhanced the binding affinity of PGC1α or LRPGC1 to NRF1/2, even under lactate stimulation (*SI Appendix*, Fig. S12 *E* and *F*).

We next analyzed the mRNA levels of downstream target genes of NRF1/2 (*Tfb1m*, *Tfb2m*, and *Tfam*). *HSP90AA1* KO and CREBBP knockdown significantly inhibited sodium lactate-induced expression of *Tfb1m*, *Tfb2m*, and *Tfam* (*SI Appendix*, Fig. S12 *G* and *H*). Moreover, the K58R, K616R, and 4 K–R mutations, as well as the S39A, S596D, and S39A/S596D mutations, suppressed sodium lactate-induced expression of *Tfb1m*, *Tfb2m*, and *Tfam* (*SI Appendix*, Fig. S12 *I* and *J*). These results indicate that HSP90α lactylation modulates its phosphorylation status, thereby promoting the binding of PGC1α or LRPGC1 to NRF1/2 after nuclear import, thereby activating the expression of NRF1/2 target genes and ultimately facilitating mitochondrial biogenesis.

### Lactate Enhances Mitochondrial Cholesterol Localization and Estrogen Production Via HSP90α Lactylation.

**Fig. 5.**
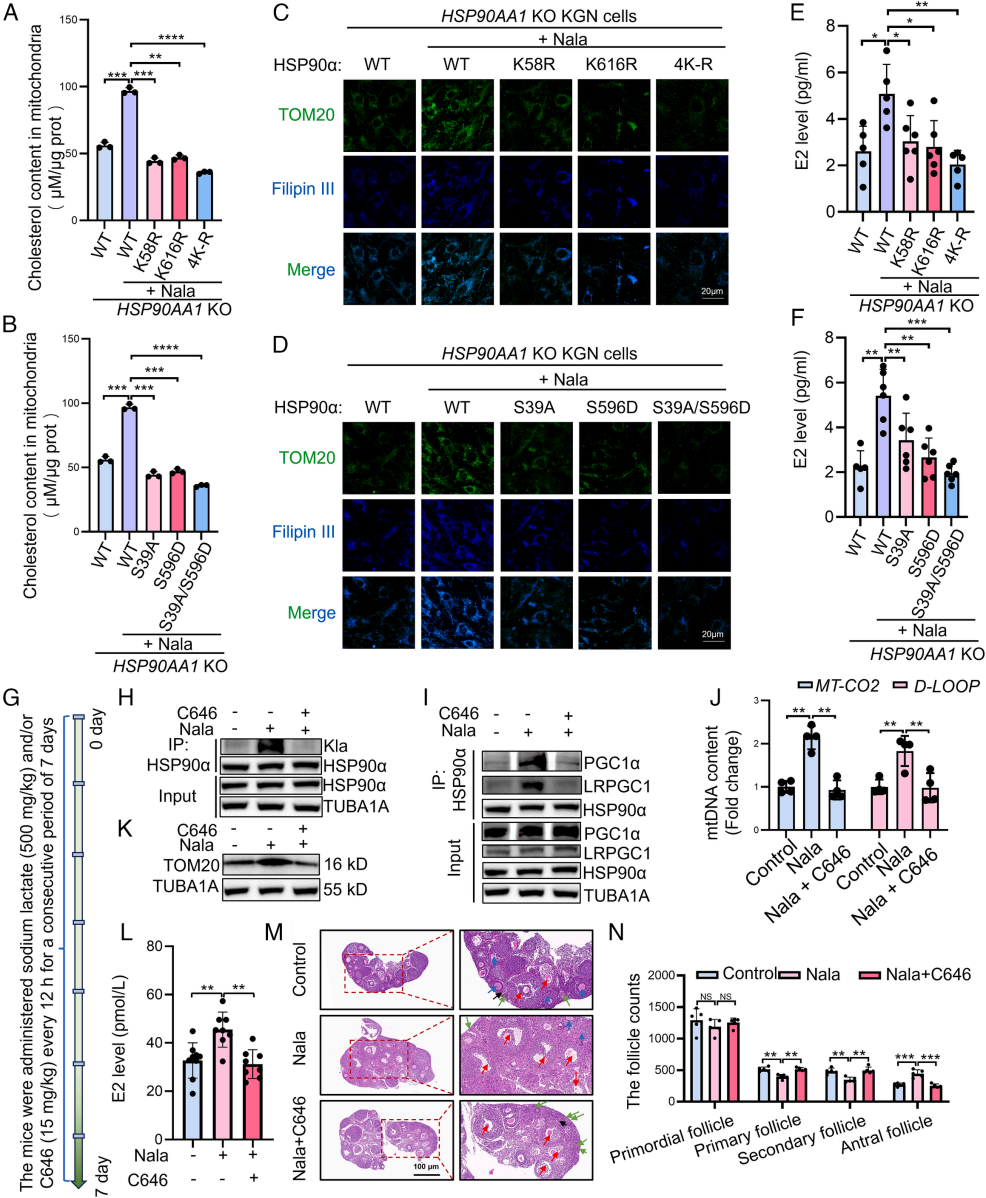
HSP90α lactylation promotes estrogen synthesis and follicular development. (*A*–*F*) Mitochondrial cholesterol content (*A* and *B*), immunofluorescence colocalization of TOM20 and cholesterol (Filipin III) (*C* and *D*), and estradiol levels (*E* and *F*) in lactate-treated *HSP90AA1* KO KGN cells expressing WT, K58R, K616R, or 4 K-R (*A*, *C*, and *E*) or expressing WT, S39A, S596D, or S39A/S596D (*B*, *D*, and *F*). (*G*) Schematic of the in vivo treatment protocol with sodium lactate and C646. (*H*–*L*) Coimmunoprecipitation analysis of HSP90α lactylation (*H*) and binding to PGC1α/LRPGC1 (*I*), RT-qPCR of mtDNA copy number (*J*), and western blot of TOM20 (*K*) in GCs; serum estradiol levels by radioimmunoassay (*L*). (*M*) H&E staining of ovarian sections from mice. (*N*) Quantification of follicle numbers at different stages from *M*. Green arrows: primordial; black: primary; blue: secondary; red: antral. Nala: sodium lactate. The data were presented as mean ± SD. Differences between groups were assessed using ANOVA. *P < 0.05; **P < 0.01; ***P < 0.001; ****P < 0.0001. NS indicates no difference.

Mitochondria both generate cellular ATP and provide the initial site for steroidogenesis in ovarian granulosa cells by converting cholesterol to pregnenolone via CYP11A1, after cholesterol is delivered to the inner mitochondrial membrane by StAR (12). To test whether HSP90α-dependent mitochondrial biogenesis affects cholesterol trafficking and estrogen synthesis, we isolated mitochondrial fractions and measured cholesterol levels. Sodium lactate treatment increased mitochondrial cholesterol content, and, as before, the mutations suppressed this effect (Fig. 5 *A* and *B*). Using TOM20 to mark mitochondria and Filipin III to detect cholesterol, immunofluorescence showed that sodium lactate treatment increased cholesterol accumulation within mitochondria (Fig. 5 *C* and *D*), the mutations suppressed this effect (Fig. 5 *C* and *D*). Concurrently, lactate-induced estrogen production was also inhibited in these mutants (Fig. 5 *E* and *F*). These data indicate that lactate enhances mitochondrial cholesterol localization and estrogen synthesis in a manner dependent on HSP90α lactylation and its downstream phosphorylation status.

To validate these findings in vivo, we administered sodium lactate to mice, with or without the CREBBP inhibitor C646 (Fig. 5*G*). Sodium lactate injection enhanced HSP90α lactylation and its binding to PGC1α/LRPGC1, whereas inhibition of CREBBP with C646 significantly suppressed the lactylation and complex formation induced by lactate (Fig. 5 *H* and *I*). Consistent with these molecular changes, lactate elevated ovarian mtDNA copy number and TOM20 protein levels, and these increases were prevented by C646 (Fig. 5 *J* and *K*). Functionally, lactate-treated mice exhibited higher estradiol levels and an increased number of antral follicles, effects that were reversed by C646 cotreatment (Fig. 5 *L*–*N* and *SI Appendix*, Fig. S13). These in vivo data suggest that HSP90α lactylation is associated with lactate-stimulated mitochondrial biogenesis and follicular development.

## Discussion

Phosphorylation of HSP90α at different sites modulates its interactions with various substrate proteins (17, 31). Although we have identified lactylation of HSP90α (21), it remains unclear whether crosstalk between its lactylation and phosphorylation coordinately regulates HSP90α–substrate interactions. Structural considerations suggest that K58 lactylation and S39 phosphorylation, both located within the N-terminal domain of HSP90α, form an integrated regulatory module (32). In contrast to the canonical model in which ULK1-mediated S39 phosphorylation destabilizes HSP90α activity and promotes client release, our data indicate that under lactate-rich conditions K58 lactylation induces localized conformational rearrangements that actively enhance ULK1 recruitment. This metabolite-driven “conformational preconditioning” alters the outcome of S39 phosphorylation, converting it from an inhibitory modification into a cooperative signal that, together with K58 lactylation, stabilizes HSP90α in a conformation favorable for client binding and nuclear translocation of PGC1α/LRPGC1. Complementing this N-terminal regulation, the middle domain of HSP90α undergoes a distinct form of structural modulation. Lactylation at K616 likely interferes with CDK5 docking through steric hindrance and charge redistribution, thereby preventing phosphorylation at S596. Because S596 phosphorylation is inhibitory for PGC1α/LRPGC1 activation, suppression of this modification effectively locks the middle domain in a conformation that supports mitochondrial client protein maturation. Together, these findings highlight how site-specific lactylation events reprogram the cross-talk between HSP90α phosphorylation and conformational dynamics, establishing a dual-site regulatory mechanism that integrates metabolic cues with chaperone function to drive mitochondrial biogenesis.

Although the histone acetyltransferases CREBBP and p300 share high structural homology and are often regarded as functionally redundant in catalyzing lysine acylation (33), accumulating evidence suggests that their roles in lactylation can be context-dependent and substrate-specific (34, 35). Our data identify CREBBP as the principal writer enzyme responsible for HSP90α lactylation under lactate-rich conditions. This specificity may reflect its unique interaction network with mitochondrial regulatory proteins in granulosa cells. In contrast, p300 has been reported as the predominant writer for other lactylation events, such as histone lactylation in macrophages during inflammatory activation (36). These observations highlight that the functional specialization within the CREBBP/p300 family is dictated by cellular environment, substrate accessibility, and upstream signaling cues. Thus, while redundancy between these enzymes is well recognized, our findings emphasize that CREBBP assumes a nonredundant role in orchestrating HSP90α-dependent mitochondrial biogenesis in the ovarian context.

Protein lactylation occurs via enzymatic and nonenzymatic pathways (37). Enzymatically, lactate is converted to lactyl-CoA by ACSS2/GTPSCS, then transferred by writer enzymes. Our findings—knockdown of ACSS2/GTPSCS reduces HSP90α lactylation and identification of CREBBP as the transferase—demonstrate that HSP90α is lactylated enzymatically under lactate-rich conditions. Alternative routes exist: alanyl-tRNA synthetase directly uses lactate and ATP to generate lactyl-AMP for transfer, and nonenzymatic reactions via intermediates like lactoyl-glutathione may also contribute to basal lactylation. Thus, cells possess a diversified lactylation landscape with multiple converging pathways, whose relative contributions require further systematic dissection.

PGC1α is a master transcriptional coactivator governing mitochondrial biogenesis and cellular energy metabolism, and its activity is tightly controlled by multiple posttranslational modifications (38–40). For example, AMPK-mediated phosphorylation enhances its transcriptional potency and stability (39), whereas GCN5-dependent acetylation suppresses its activity (40), and SIRT1-mediated deacetylation is required for both initiation and maintenance of its transcriptional programs (41). LRPGC1, a recently identified splicing isoform of PGC1α (14), has not yet been reported to undergo such modifications, leaving its regulation largely unexplored. Our findings demonstrate that sodium lactate does not directly modify PGC1α/LRPGC1; instead, lactylation of HSP90α governs their nuclear translocation by reprogramming chaperone interactions. This raises the important question of whether the observed effects are entirely mediated through altered chaperone-client dynamics, or whether changes in HSP90α conformation secondarily influence PGC1α’s own PTM landscape, thereby modulating its activity and stability. Systematic mapping of phosphorylation, acetylation, and other modifications on PGC1α/LRPGC1 under lactate-rich conditions will be essential to determine the extent to which chaperone-dependent nuclear import intersects with direct coactivator regulation.

As one of the most abundant molecular chaperones, HSP90 accounts for approximately 1 to 2% of total cellular proteins under physiological conditions and can increase to 4 to 6% during stress responses (42). The HSP90 family comprises two highly conserved cytosolic isoforms, HSP90α and HSP90β, both of which are indispensable for essential cellular processes including protein folding (43), maturation of signaling proteins (44), cell growth (45), and stress adaptation (46). HSP90α is stress-inducible and subject to extensive regulation by posttranslational modifications (16, 31, 47), whereas HSP90β is constitutively expressed and provides basal chaperone function (48). Our findings demonstrate that sodium lactate induces site-specific lactylation at K58 and K616 of HSP90α, which in turn differentially regulates phosphorylation at S39 and S596, these results reveal a regulatory mechanism for this inducible isoform. Nevertheless, whether HSP90β or other molecular chaperones can also undergo lactylation and compensate for the loss of HSP90α activity remains an important unresolved question. Given reports that HSP90 isoforms can partially substitute for one another in maintaining proteostasis and stress resistance, it is conceivable that a broader chaperone network may contribute to lactate-mediated mitochondrial regulation. Thus, future studies should examine lactylation status across HSP90 isoforms and assess their relative capacity to modulate PGC1α/LRPGC1 function and mitochondrial biogenesis in ovarian cells.

Our findings indicate that activation of the lactate–HSP90α–PGC1α/LRPGC1 axis drives mitochondrial mass expansion and basal respiratory activity. However, these metrics reflect quantity rather than functional integrity. A thorough assessment requires quality parameters—respiratory control ratio, ATP production, membrane potential, ROS, and mitophagic flux—to confirm that newly generated mitochondria are both abundant and competent.

Several limitations of this study should be acknowledged. First, although our findings establish a causal role for HSP90α lactylation in regulating PGC1α/LRPGC1 nuclear translocation and mitochondrial biogenesis, the physiological relevance of these modifications requires in vivo validation using conditional, ovary-specific knock-in models that harbor K58R and K616R substitutions or targeted deletion of *Crebbp*. Such genetically precise systems would provide definitive evidence for the role of HSP90α lactylation in ovarian function under both physiological and pathological conditions. Second, the mechanistic basis by which K58 and K616 lactylation remodel HSP90α conformation and client interactions remains incompletely understood. High-resolution structural studies, such as cryoelectron microscopy or crystallography of HSP90α in complex with CREBBP, ULK1, or CDK5, are indispensable for visualizing how these modifications reshape interaction interfaces and phosphorylation outcomes. Finally, it remains uncertain whether this lactylation-dependent mechanism is unique to granulosa cells or represents a more general principle in steroidogenic tissues such as the adrenal cortex or Leydig cells. Addressing these limitations are critical for fully delineating the scope of lactylation in cellular metabolic regulation and reproductive physiology.

In summary, we reveal a previously unrecognized pathway by which lactate influences mitochondrial biogenesis through site-specific lactylation of HSP90α (*SI Appendix*, Fig. S14). We show that lactylation at K58 and K616 differentially modulates HSP90α phosphorylation, thereby facilitating nuclear translocation of PGC1α/LRPGC1 and activation of NRF1/2-dependent transcriptional programs. This lactate–HSP90α interaction establishes a mitochondrial regulatory axis that enhances estradiol biosynthesis and supports follicular development.

## Materials and Methods

### Cell Culture and Treatment Protocols.

KGN cells were cultured in DMEM-F12 medium (Gibco), and HEK-293 T cells were cultured in DMEM medium (Gibco) supplemented with 10% fetal bovine serum and 1% penicillin-streptomycin. Both cell lines were incubated at 37 °C in a humidified atmosphere containing 5% CO_2_. For pharmacological treatments, cells were pretreated with 50 nM 17-AAG, 3 mM α-CHCA, and 200 ng/mL LPS for 2 h prior to 12 h sodium lactate exposure.

Additional materials and methods can be found in *SI Appendix*, *Materials and Methods*.

## Supplementary Material

Appendix 01 (PDF)

Dataset S01 (XLS)

## Data Availability

All study data are included in the article and/or *SI Appendix*.
