# Supplementary material for: HSP90α lactylation orchestrates PGC1α and LRPGC1 nuclear translocation driving mitochondrial biogenesis
Source: Proc Natl Acad Sci U S A. 2026 Jul 21;123(30):e2528979123. doi: 10.1073/pnas.2528979123 (PMC13416674; doi:10.1073/pnas.2528979123)
Supplement: Supplementary file 1 — Appendix 01 (PDF) [file pnas.2528979123.sapp.pdf]

Supporting Information Text

Materials and Methods

Mice

Mice aged 3-4 weeks were randomly divided into three groups: control group, sodium lactate injection group, and sodium lactate + C646 co-injection group. The control group received intraperitoneal injections of saline every 12 h; the sodium lactate group was injected with sodium lactate (500 mg/kg) every 12 h; and the combination group received concurrent injections of sodium lactate (500 mg/kg) and C646 (15 mg/kg) at the same interval. The treatment regimen was continued for 7 consecutive days. After the treatment, ovarian tissues, serum, and granulosa cells were collected for subsequent experimental analyses. Throughout the experiment, all mice were maintained under a 12-h light/12-h dark cycle with ad libitum access to food and water.

Reagents and antibodies

All reagents and antibodies used in this study were obtained from commercial sources as follows: TOM20 (#42406S), HSP60 (#12165S), CREBBP (#7389S), phosphorylation antibody (#9631S), ULK1 (#8054T), HIS-tag antibody (#2365S), and Histone H3 (#14269) were purchased from Cell Signaling Technology. TUBA1A (#66031-1-Ig), Flag-tag antibody (#80801-2-RR), HSP90α (#60318-1-Ig), CDK5 (#10430-1-AP), GST-tag antibody (#66001-2-Ig), ACAT1 (#16215-1-AP), PAFAH1B3 (#20564-1-AP), DLAT1 (#13426-1-AP), LPCAT3 (#67882-1-lg) and HA-tag antibody (#66006-2-Ig) were obtained from Proteintech. PGC1α (#1061135-38) and NRF1 (#AB175932) were from ABcam. LRPGC1 (#SC-518025) was acquired from Santa Cruz Biotechnology. HSP70 (#bs-0244R) and HSP40 (#bsm-33432M) were purchased from Bioss. Pan-Kla antibody (#PTM-1401RM) was from PTMAL. NRF2 (#ZF4372571A) was supplied by Invitrogen. Horseradish peroxidase (HRP)-conjugated goat anti-mouse IgG (#ab6789) and HRP-conjugated goat anti-rabbit IgG (#ab6721) were from Abcam. Alexa Fluor 488 AffiniPure goat anti-mouse IgG (#ZF-0512) and Rhodamine AffiniPure goat anti-rabbit IgG (#ZF-0316) were from ZSGB-

BIO. Alexa Fluor 488 AffiniPure goat anti-rabbit IgG (#33107ES60) and Rhodamine AffiniPure goat anti-mouse IgG (#33206ES60) were purchased from YESEN. ACAA1B (#A7422), ACAA2 (#A1399), PRDX6 (#A4286), AARS1 (#A15017) were acquired from ABclonal. NAT10 (#R015057), SCP2 (#R015147), FASN (#R013857), HADHB (#P106623), HADHA (#R013093), ACAA2 (#R014299), HSP90β (#M011178) were acquired from epizyme. AZD3965 (#S7339), C646 (#S7152), and 4-Chloro-α-cyanocinnamic acid (α-CHCA; #S8612) were obtained from Selleck Chemicals. LPS (#HY-D1056) and 17AAG (#HY-10211) were purchased from MCE. Filipin III (#MX5213) were obtained from Merck. Sodium L-lactate (#71718) was acquired from Sigma-Aldrich. Lactyl-CoA (ATXB-00809) was purchased from TargetMol.

Western Blot Analysis

Cellular proteins were extracted using lysis buffer (Beyotime) containing protease inhibitor PMSF (Beyotime), with protein concentrations quantified by BCA assay (Beyotime). Equal protein quantities (15 μg/lane) were resolved by 10% SDS-PAGE and electrotransferred to PVDF membranes (Millipore). After 2 h blocking with 5% BSA at room temperature, membranes were probed with primary antibodies overnight at 4°C. Following three TBST washes, HRP-conjugated secondary antibodies were applied for 2 h at room temperature. Protein signals were developed using ECL HRP substrate (Advansta) and quantified by densitometry with TUBA1A as the loading control.

Quantitative Real-Time PCR Analysis

Total RNA was isolated using TRIzol reagent (Invitrogen) and reverse transcribed into cDNA with PrimeScript™ RT Master Mix (Takara). qPCR was performed using Hieff UNICON® SYBR Green Master Mix (Vazyme) on an ABI StepOnePlus™ system (Applied Biosystems), with gene-specific primers (sequences provided in Supplementary Table S1). Following amplification, reaction specificity was confirmed by melting curve analysis. Relative gene expression was calculated using the $2^{(-\Delta\Delta Ct)}$

method with Tuba1a as the endogenous control.

RNA Interference Protocol

Gene-specific siRNAs targeting PGC1α, LRPGC1, and HSP90AA1 were purchased from GenePharma (Shanghai, China), while siRNAs against NAT10, CREBBP, DLAT, SCP2, ACAA1B, ACAT1, PAFAH1B3, FASN, ACAT3, LPCAT3, HADHB, PRDX6, HADHA, ACAA2, and AARS1 were obtained from GeneRay. The list was shown in Supplementary table 2. All siRNA transfections were performed using Lipofectamine 3000 reagent (Invitrogen) according to the manufacturer's protocol.

Plasmid Construction

The plasmids of wild-type HSP90α and its mutants (K58R, K284R, K568R, K616R, 4K–R, S39A, S596D, the double mutant S39A/S596D, S39D, S596A, and the double mutant S39D/S596A) with an N-terminal Flag tag were constructed into the pcDNA3.1 vector. Meanwhile, PGC1α with a Flag tag, as well as LRPGC1α with both HA and EGFP tags, were also cloned into the pcDNA3.1 vector. In addition, His-tagged prokaryotic expression plasmids of HSP90α, including the wild-type and 4K–R mutants, were constructed into the pET-28a vector. Meanwhile, the prokaryotic expression plasmids of GST-tagged PGC1, LRPGC1, ULK1, CDK5 and the CREBBP HAT domain were constructed into the pGEX-4T-1 vector. All plasmids were constructed by Tsingke Biotechnology Co.

Coimmunoprecipitation Assay

Cells were lysed in ice-cold IP lysis buffer (Beyotime) supplemented with protease inhibitor PMSF (Beyotime, #ST506-2). Cell lysates were incubated with target-specific antibodies overnight at 4°C, followed by addition of 25 μl Protein A/G magnetic beads (Thermo Fisher Scientific, #88803) for 1 h at 4°C. After extensive washing, immunoprecipitated complexes were analyzed by Western blotting using relevant antibodies.

CRISPR-Cas9-Mediated *HSP90AA1* Knockout Generation

The *HSP90AA1* knockout cell line was generated using CRISPR-Cas9 technology (Cyagen Company). Briefly, KGN cells were transiently transfected with a HSP90AA1-targeting gRNA expression plasmid (target sequence: GATCTGTCAAGCTTTCATAC-CGG). Following transfection, single-cell clones were isolated by limiting dilution in 96-well plates. Successful knockout clones were identified through PCR screening and validated by Sanger sequencing.

Measurement of Lactyl-CoA concentration

Lactyl-CoA concentrations in KGN cells were quantified using a commercial Lactyl-CoA ELISA Kit (#FT-PR65405) according to the manufacturer's protocol. Absorbance at 450 nm was measured with a TECAN microplate reader, and sample concentrations were determined by extrapolation from a standard curve generated with the provided reference standards.

Mitochondrial Staining with MitoTracker Green

KGN cells cultured on coverslips were treated as specified, followed by co-staining with 50 nM MitoTracker Green and 20 nM TMRM for 30 min at 37°C. Fluorescence imaging was performed using a Zeiss LSM 710 META confocal microscope, with subsequent quantification of fluorescence intensity conducted in ImageJ (version 1.42q).

Quantification of Mitochondrial DNA Content

Total cellular DNA was isolated using the QIAamp DNA Mini Kit (#4991108) according to the manufacturer's instructions. Mitochondrial DNA (mtDNA) content was determined by quantitative real-time PCR (qPCR) through comparative analysis of mitochondrial genes (D-loop region and MT-CO2) normalized to the nuclear-encoded β-actin gene. Primer sequences are detailed in Supplementary Table S3.

OCR analysis

Mitochondrial respiration and glycolytic flux in KGN cells were simultaneously measured using a Seahorse XF96 Extracellular Flux Analyzer. Cells were seeded at densities of 4×10^4 or 8×10^4 cells per well in 96-well assay plates pre-coated with Cell-Tak coating reagent (Corning). Following a 1 h equilibration in XF RPMI substrate-enriched medium (phenol red-free, supplemented with 10 mM glucose, 2 mM glutamine, and 1 mM sodium pyruvate), mitochondrial function was assessed using the Cell Mito Stress Test Kit (Agilent). The oxygen consumption rate (OCR) was dynamically monitored by sequential injections of 180 μL of assay medium, followed by 20 μL each of oligomycin (2 μM), carbonyl cyanide-4-(trifluoromethoxy)phenylhydrazone (FCCP, 2 μM), and a rotenone/antimycin A cocktail (0.5 μM). All metabolic parameters were normalized to cell counts for comparative analysis.

Identification of HSP90α Lactylation Writers via HPLC-MS/MS Screening

Specific anti-HSP90α antibody was used to immunoprecipitate HSP90α-associated protein complexes, followed by compositional characterization using high-performance liquid chromatography-tandem mass spectrometry (HPLC-MS/MS). To systematically identify potential regulators of HSP90α lactylation, we performed correlation analysis between HSP90α-interacting proteins and known acyltransferases, thereby identifying specific acyltransferases that interact with HSP90α.

Identification of lactylation modification on HSP90α via LC-MS/MS analysis

The HSP90α lactylation was performed in collaboration with Jingjie PTM BioLabs (Hangzhou, China). Protein samples underwent reduction with 5 mM dithiothreitol (56°C, 30 min) followed by alkylation using 11 mM iodoacetamide (room temperature, 15 min, dark conditions). After dilution with 100 mM TEAB to reduce urea concentration below 2 M, proteins were digested sequentially with trypsin using two-step enzymatic cleavage: an overnight digestion at 1:50 (trypsin: protein) ratio followed by a 4 h secondary digestion at 1:100 ratio. The resulting peptides were purified using C18 solid-phase extraction columns.

For modification-specific enrichment, tryptic peptides were resuspended in NETN buffer (100 mM NaCl, 1 mM EDTA, 50 mM Tris-HCl, 0.5% NP-40, pH 8.0) and immunoprecipitated with specific antibody-conjugated beads overnight at 4°C with gentle agitation. After extensive washing with NETN buffer and water, bound peptides were eluted using 0.1% trifluoroacetic acid, pooled, and lyophilized.

LC-MS/MS analysis was conducted using a nanoElute UHPLC system (Bruker Daltonics) equipped with a custom-made reversed-phase column (25 cm × 100 μm). Peptides were separated and analyzed by timsTOF Pro mass spectrometer (Bruker Daltonics) operating in PASEF mode. Acquired MS/MS spectra were processed through MaxQuant (v1.6.6.0) against the SwissProt mouse database (20,366 entries) with reverse decoy database for FDR control (set at <1%). Lysine lactylation sites were identified with localization probability >0.75. Quantitative comparison of modified peptides was performed by intensity-based absolute quantification, with normalized ratios calculated against corresponding protein expression levels from duplicate experimental runs.

Immunofluorescence Staining Procedure

KGN cells cultured on coverslips were fixed with 4% paraformaldehyde for 15 min at room temperature. Following fixation, cells were permeabilized with 0.05% Triton X-100 in PBS for 10 min at 4°C and subsequently blocked with 5% bovine serum albumin (BSA) in PBS for 2 h at room temperature. The samples were then incubated overnight at 4°C with primary antibodies diluted in 1% BSA. After three PBS washes, cells were incubated for 2 h in the dark with fluorophore-conjugated secondary antibodies, followed by another three PBS washes. Nuclei were counterstained with DAPI (#KGA215-10) for 10 min before imaging with a Zeiss LSM 900 laser scanning confocal microscope.

Analysis of Cholesterol Colocalization with Mitochondria

First, cells were incubated with a primary antibody against the mitochondrial outer membrane protein TOM20, followed by detection with a fluorophore-conjugated

secondary antibody to specifically label the mitochondrial network. Second, cells were treated with 50 nM Filipin III (#MX5213) at room temperature for 60 min to specifically visualize cholesterol distribution. Finally, the colocalization of cholesterol and mitochondria was observed and analyzed using fluorescence microscopy.

Measurement of Mitochondrial Cholesterol Content

According to the manufacturer's instructions, mitochondria were isolated using a Mitochondria Extraction Kit (#C3601), followed by the extraction and quantification of mitochondrial cholesterol using a Cholesterol Extraction Kit (#S0211S).

^{125}I-labeled Estradiol Radioimmunoassay

A competitive binding radioimmunoassay (RIA) system (Beijing North Biotechnology Research Institute, China, #B05PZB) was employed to quantify serum estradiol (E2) concentrations in accordance with the manufacturer’s instructions. The assay is based on the principle that endogenous E2 competes with exogenously added ^{125}I-labeled estradiol (^{125}I-E2) for a limited number of specific antibody binding sites. Test samples and calibrators were combined with ^{125}I-E2 tracer and anti-estradiol polyclonal antiserum in coated tubes. After incubating at 37 °C for 90 min to allow the competitive immunoreaction to reach equilibrium, an immunoseparation reagent (PR) was introduced to precipitate the antibody-bound fraction. The mixture was centrifuged at 3,600 × g for 20 min to separate bound from free tracer. The radioactivity in the pellet, representing the antibody-bound complex, was measured using a gamma counter. A standard curve was constructed by plotting the percentage of bound radioactivity against known E2 concentrations in the calibrators, and the E2 levels in unknown samples were determined by interpolation from this curve.

Subcellular Fractionation for Nuclear and Cytoplasmic Protein Extraction

Nuclear and cytoplasmic protein fractions were isolated from cells using the NE-PER Nuclear and Cytoplasmic Extraction Reagents (Thermo Fisher Scientific, Waltham, MA, USA; Cat: #78833) according to the manufacturer's instructions. The resulting

cytosolic and nuclear extracts were aliquoted and stored at -80°C for subsequent analysis by western blotting or immunoprecipitation.

Structural Modeling and Molecular Docking Analysis

The amino acid sequence of HSP90α was retrieved from the UniProt database, and its tertiary structure was predicted using AlphaFold 3. Due to the incomplete experimentally resolved structure of the HSP90α protein, structural modeling was performed via AlphaFold 3 as follows: the amino acid sequence was submitted to the AlphaFold 3 server, and three-dimensional structure prediction was carried out using default parameters. Model quality was evaluated using the predicted TM-score (pTM), which ranges from 0 to 1, with values closer to 1 indicating higher confidence in the predicted structure and greater predictive accuracy. The resulting wild-type HSP90α model achieved a pTM value of 0.79, demonstrating high reliability and predictive accuracy, making it suitable for subsequent mutational analysis. On this basis, site-directed mutagenesis was performed to replace lysine (K) residues at positions 58, 284, 568, and 616 with arginine (R), followed by energy minimization to optimize the spatial conformation of the mutant proteins. Subsequently, lactylation modifications at lysine residues 58 and 616 were introduced using the CHARMM-GUI platform, where the lysine side chains were replaced with lactate-group-derived structures to establish correct bond linkages and atom types. After residue modification, the protein structures were subjected to hydrogenation and side-chain conformation optimization, ultimately generating lactylation-modified protein structure files in PDB format for subsequent molecular docking calculations. Molecular docking was then performed between wild-type, mutant, and lactylation-modified HSP90α and the proteins PGC1α, LRPGC1, ULK1, CDK5, and CBP. Based on the top-ranked docking poses (Top1), the binding modes of the following protein complexes were determined: HSP90α-PGC1α, HSP90α-LRPGC1, HSP90α-ULK1, HSP90α-CDK5, and HSP90α-CBP.

Protein Purification Protocol

Expression constructs containing His-tagged wild-type HSP90α or its mutant (4K–R),

along with GST-tagged PGC1α, LRPGC1, ULK1, CDK5 and the CREBBP HAT domain, were separately transformed into Escherichia coli BL21(DE3) competent cells. Bacterial cultures were grown in LB medium to an OD600 of approximately 0.6 before inducing protein expression with 0.5 mM IPTG at 16°C overnight (16 h). Cells were harvested by centrifugation (8,000×g, 10 min, 4°C), resuspended in chilled PBS, and lysed via ultrasonication (300 W output with 3 sec pulses and 5 sec intervals for 15 min total) on ice. Following clarification at 12,000×g for 30 min, the soluble fractions were purified using either Ni NTA Magarose Beads (#SM008005) for His-tagged proteins or Glutathione Magarose Beads (#SM002005) for GST-fusion proteins.

In vitro lactylation assay

We performed in vitro lactylation of HSP90α using two different methods. In the first method, HEK-293T cells were overexpressed with Flag-tagged CREBBP, and the Flag-CREBBP protein was immobilized using Flag magnetic beads. Purified His-tagged HSP90α and 100 μM Lactyl-CoA were added to the bead/ protein complex in a reaction buffer consisting of 25 mM Tris-HCl (pH 8.0), 150 mM NaCl, 10% glycerol, 1 mM sodium butyrate, and 1 mM DTT. The reaction was carried out at 37°C for 30 minutes, after which the lactylation level of HSP90α was detected. In the second method, His-tagged HSP90α and the GST-tagged HAT domain of CREBBP were purified separately. The GST-CREBBP HAT and His-HSP90α were then incubated together with 100 μM Lactyl-CoA in the same reaction buffer as described above at 37°C for 30 minutes, followed by detection of HSP90α lactylation.

Pull-down Assay for Protein-Protein Interaction Analysis

First, the purified His-tagged wild-type HSP90α protein was subjected to in vitro lactylation modification reaction. Subsequently, the purified His-tagged wild-type HSP90α, lactylated HSP90α, and lactylation-deficient mutant HSP90α were individually incubated with GST-tagged PGC1α, LRPGC1, ULK1, CDK5 or CREBBP proteins at 4°C for 2 h, followed by GST pull-down assays. After the pull-down reactions, the captured protein complexes were resolved by SDS-PAGE and then

analyzed by Western blotting using anti-His and anti-GST specific antibodies to verify the protein-protein interactions.

Statistical Analysis

Statistical analyses were conducted using GraphPad Prism 7 (GraphPad Software, San Diego, CA). Data are expressed as mean ± standard deviation (SD) from at least three independent biological replicates. Intergroup comparisons were performed by one-way ANOVA with post-hoc LSD tests. Statistical significance was defined as $p < 0.05$.

Lead Contact and Resource Availability

Requests for materials and additional information should be addressed to the Lead Contact, Dr. Gang Wu (2022205011@stu.njau.edu.cn).

Ethical Compliance

All animal experiments were performed following the Nanjing Agricultural University Animal Care Committee guidelines (Nanjing, China) and in compliance with established international standards for laboratory animal welfare.

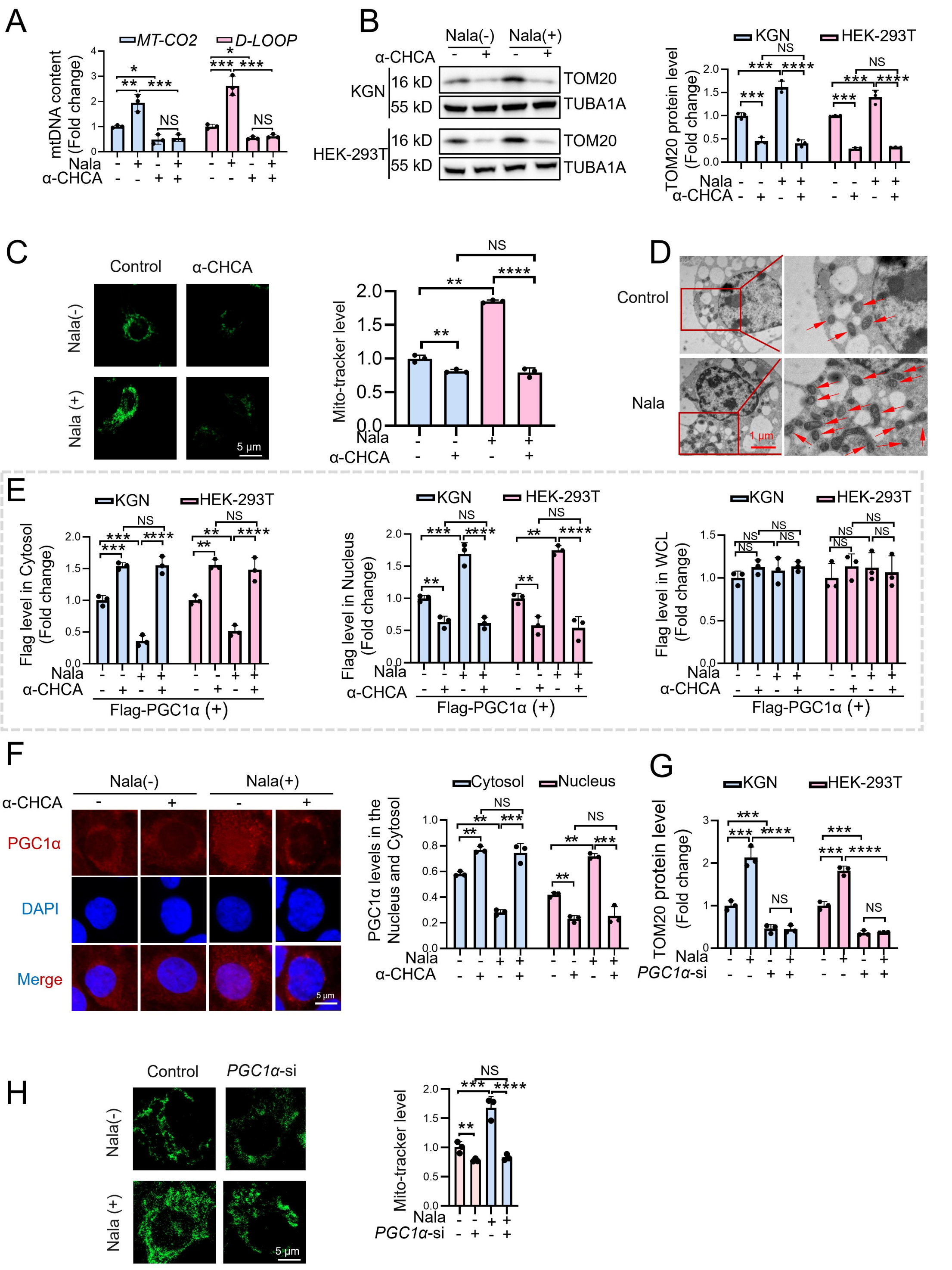

Fig S1. Lactate-induced mitochondrial biogenesis via PGC1α

A mtDNA copy number (*MT-CO2* and *D-Loop*) in KGN cells pretreated with α-CHCA followed by sodium lactate (Nala). **B** TOM20 protein levels (western blot) in KGN and HEK-293T cells treated as in A. **C** Mitochondrial content assessed by MitoTracker staining and quantitative fluorescence microscopy in KGN cells treated as in A. Scale bar = 5 μm. **D** Mitochondrial morphology and number assessed by transmission

electron microscopy in KGN cells treated with 15 mM sodium lactate for 12 h. Scale bar = 1 μm. **E** Quantification of Flag-PGC1α protein levels in cytoplasmic, nuclear, and whole-cell lysate (WCL) fractions (corresponding to Fig. 1A). **F** Subcellular localization of PGC1α by immunofluorescence in KGN cells pretreated with α-CHCA followed by sodium lactate. Scale bar = 5 μm. **G** Quantification of TOM20 protein levels from Fig. 1C, normalized to TUBA1A. **H** Mitochondrial content (MitoTracker) in KGN cells transfected with PGC1α siRNA followed by sodium lactate treatment. Nala: Sodium lactate. The data were presented as mean ± SD. Differences between groups were assessed using one-way analysis of variance (ANOVA). *$P < 0.05$; **$P < 0.01$; ***$P < 0.001$; ****$P < 0.0001$. NS indicates no difference.

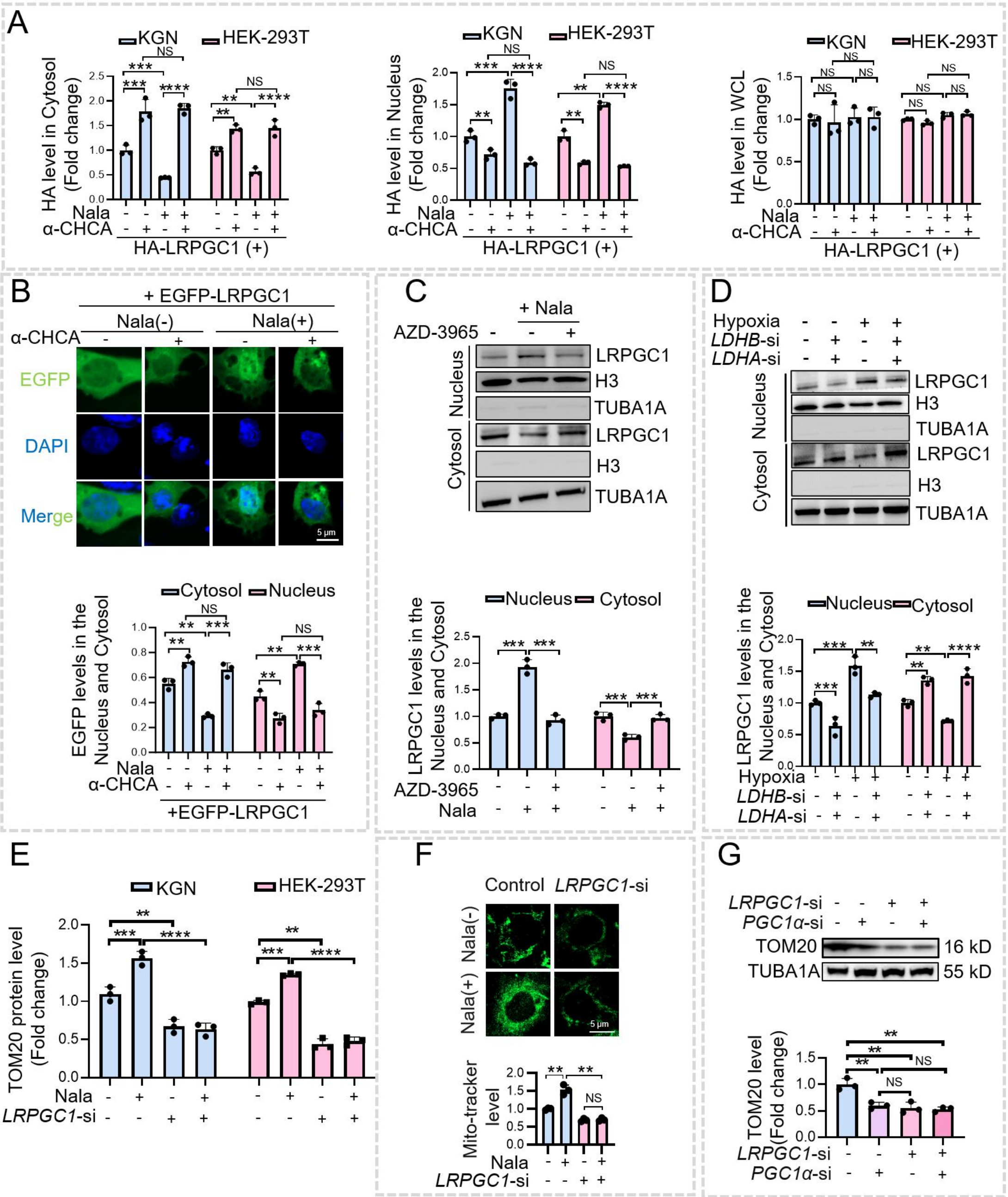

Fig S2. Lactate-induced mitochondrial biogenesis via LRPGC1

A Quantification of HA-LRPGC protein levels in cytoplasmic, nuclear, and whole-cell lysate (WCL) fractions (corresponding to Fig. 1E). **B** Immunofluorescence analysis of EGFP-LRPGC1 localization in KGN cells treated with α-CHCA and sodium lactate. Scale bar = 5 μm. **C** Western blot analysis of LRPGC1 distribution in nuclear and cytoplasmic fractions from KGN cells pretreated with AZD-3965 (MCT1 inhibitor) followed by sodium lactate. **D** Western blot analysis of LRPGC1 distribution in nuclear and cytoplasmic fractions from KGN cells transfected with *LDHA/B* siRNAs followed

by hypoxic treatment (1% O_2). **E** Quantification of TOM20 protein levels from Fig. 1G. **F** Mitochondrial content (MitoTracker) in KGN cells transfected with LRPGC1 siRNA followed by sodium lactate treatment. **G** The protein levels of TOM20 were detected in KGN cells following knockdown of PGC1α, LRPGC1, or both using specific siRNAs. Nala: Sodium lactate. The data were presented as mean ± SD. Differences between groups were assessed using one-way analysis of variance (ANOVA). *$P < 0.05$; **$P < 0.01$; ***$P < 0.001$; ****$P < 0.0001$. NS indicates no difference.

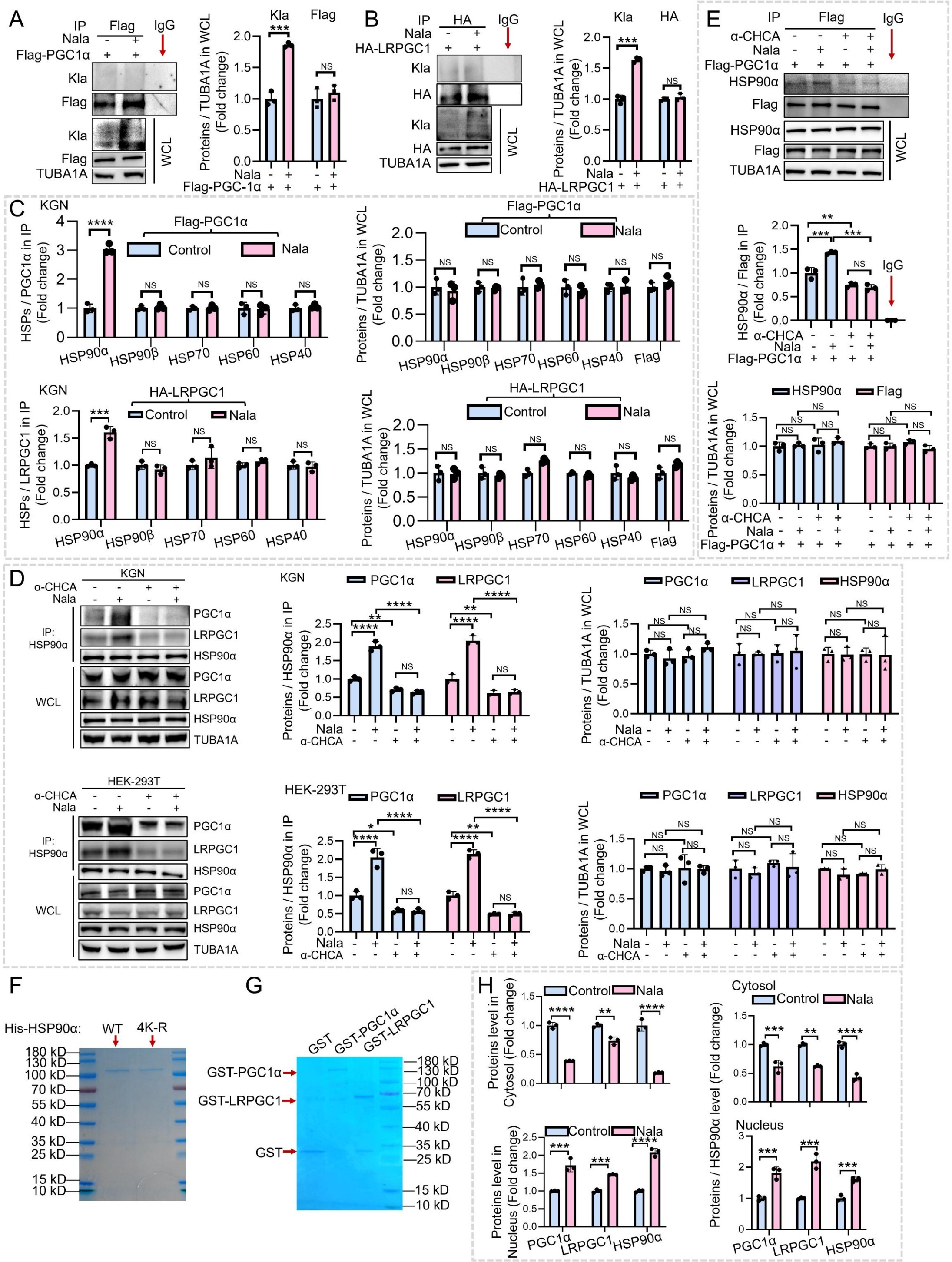

Fig S3. Inhibition of lactate production blocks its binding to PGC1α or LRPGC1.

A Coimmunoprecipitation analysis of PGC1α lactylation in sodium lactate-treated KGN cells. **B** Coimmunoprecipitation analysis of LRPGC1 lactylation in sodium lactate-treated KGN cells. **C** Quantification of binding between PGC1α or LRPGC1 and HSP family proteins, and HSP family expression levels (from Fig. **1I, 1J**). **D** Coimmunoprecipitation using HSP90α antibody to assess its binding to PGC1α and

LRPGC1 in KGN and HEK-293T cells pretreated with α-CHCA followed by sodium lactate. **E** Coimmunoprecipitation analysis of HSP90α interaction with Flag-PGC1α in KGN cells pretreated with α-CHCA then sodium lactate. **F** SDS-PAGE of purified HSP90α WT and 4K–R. **G** SDS-PAGE of purified GST-PGC1α and GST-LRPGC1. **H** Quantification of PGC1α, LRPGC1, and HSP90α levels in cytoplasmic and nuclear fractions, and their binding interactions within each fraction (from Fig. **1M**). Nala: Sodium lactate. The data were presented as mean ± SD. Differences between groups were assessed using one-way analysis of variance (ANOVA). *$P < 0.05$; **$P < 0.01$; ***$P < 0.001$; ****$P < 0.0001$. NS indicates no difference.

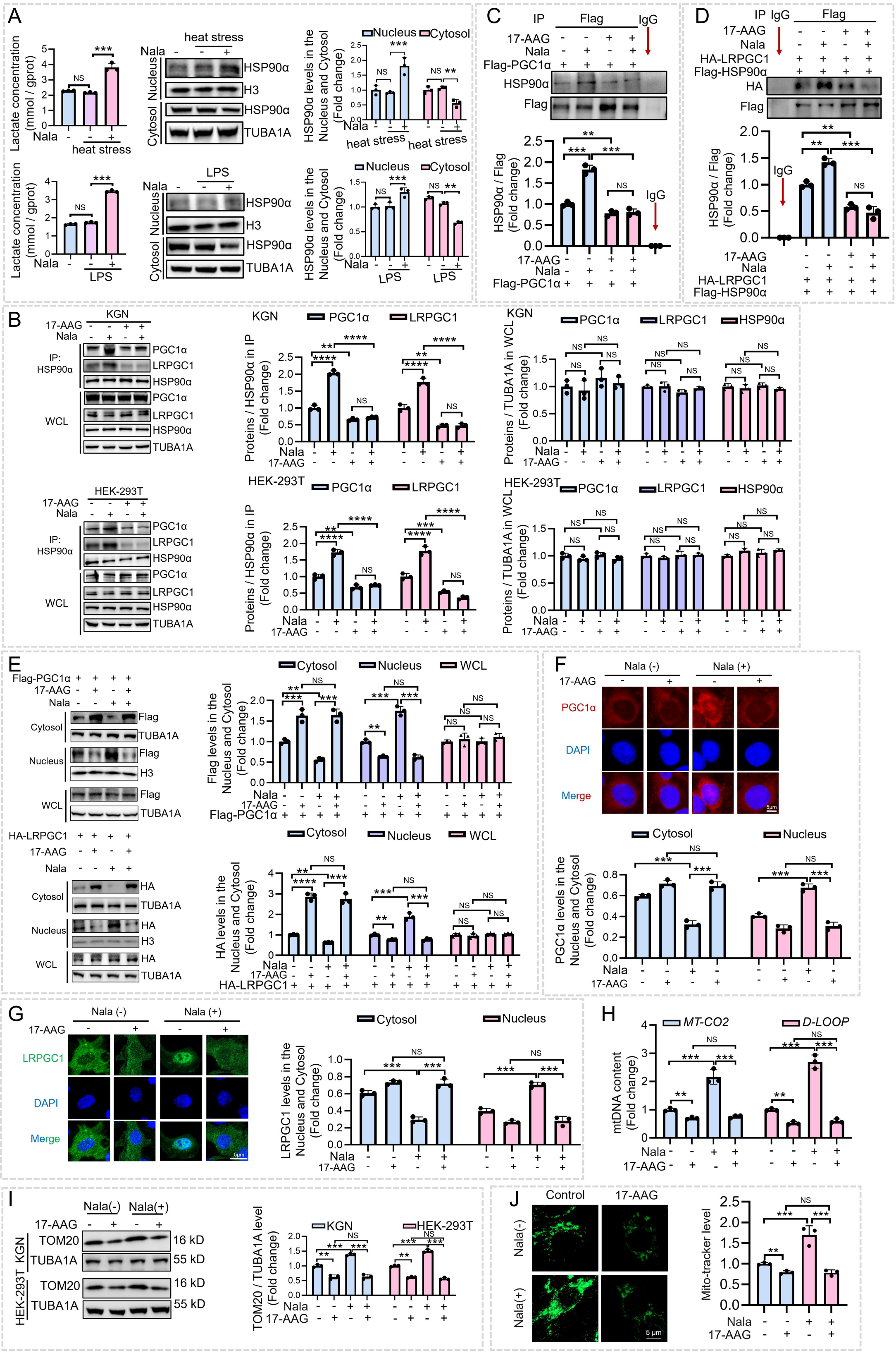

Fig S4. Inhibition of HSP90α activity blocks its binding to PGC1α or LRPGC1.

A Analysis of intracellular lactate levels and HSP90α nucleocytoplasmic distribution in HEK-293T cells treated with heat stress or LPS in the presence of sodium lactate. **B**

Coimmunoprecipitation analysis of HSP90α binding to PGC1α and LRPGC1 in KGN and HEK-293T cells pretreated with 17-AAG followed by sodium lactate. **C** Coimmunoprecipitation analysis of PGC1α-HSP90α complex formation in KGN cells transfected with Flag-PGC1α, pretreated with 17-AAG, and then stimulated with sodium lactate. **D** Coimmunoprecipitation analysis of LRPGC1-HSP90α complex formation in KGN cells co-transfected with HA-LRPGC1 and Flag-HSP90α, followed by 17-AAG pretreatment and sodium lactate stimulation. **E** Nuclear and cytoplasmic fractionation analysis of Flag-PGC1α and HA-LRPGC1 distribution in KGN cells treated with 17-AAG and sodium lactate. **F** Immunofluorescence analysis of PGC1α subcellular localization in KGN cells pretreated with 17-AAG followed by sodium lactate. Scale bar = 5 μm. **G** Immunofluorescence analysis of LRPGC1 subcellular localization in KGN cells treated with 17-AAG and sodium lactate. Scale bar = 5 μm. **H** RT-qPCR analysis of mtDNA copy number in KGN cells pretreated with 17-AAG followed by sodium lactate. **I** Western blot analysis of TOM20 protein levels in KGN cells pretreated with 17-AAG followed by sodium lactate. **J** Mitochondrial content assessed by MitoTracker staining and quantitative fluorescence microscopy. Scale bar = 5 μm. Nala: Sodium lactate. The data were presented as mean ± SD. Differences between groups were assessed using one-way analysis of variance (ANOVA). *$P < 0.05$; **$P < 0.01$; ***$P < 0.001$; ****$P < 0.0001$. NS indicates no difference.

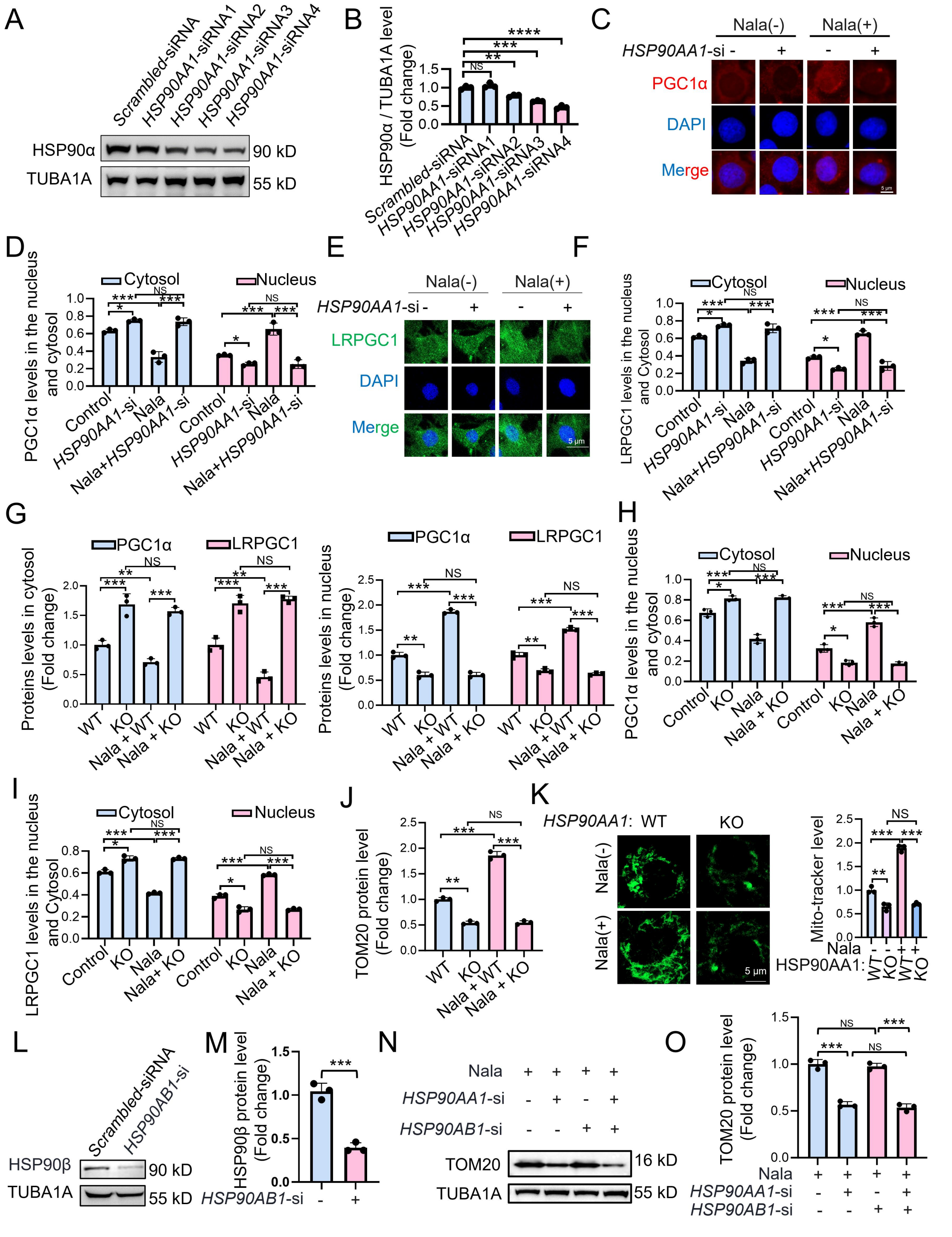

Fig S5. Knockdown of HSP90α inhibited lactate-induced mitochondrial biogenesis

A Western blot validation of HSP90α knockdown efficiency in KGN cells. **B** Quantitative analysis of HSP90α protein levels in A. **C** Immunofluorescence analysis of PGC1α subcellular localization in HSP90α-knockdown KGN cells treated with sodium lactate. **D** Quantitative analysis of PGC1α fluorescence intensity in C. **E** Immunofluorescence analysis of LRPGC1 subcellular localization in HSP90α-

knockdown KGN cells treated with sodium lactate. **F** Quantitative analysis of LRPGC1 fluorescence intensity in E. **G** Quantification of PGC1α, LRPGC1, and HSP90α levels in cytoplasmic/nuclear fractions (from Fig. 1O). **H** Quantitative analysis of PGC1α fluorescence intensity (from Fig. 1P). **I** Quantitative analysis of LRPGC1 fluorescence intensity (from Fig. 1Q). **J** Quantification of TOM20 protein levels (from Fig. 1S). **K** Mitochondrial content assessed by MitoTracker staining in *HSP90AA1* KO KGN cells treated with sodium lactate. Scale bar = 5 μm. **L** Western blot validation of HSP90β knockdown efficiency in KGN cells. **M** Quantitative analysis of HSP90β protein levels in L. **N** Western blot analysis of TOM20 protein levels in KGN cells knocked down for HSP90α, HSP90β, or both, followed by sodium lactate treatment. **O** Quantitative analysis of TOM20 protein levels in N. Nala: Sodium lactate. The data were presented as mean ± SD. Differences between groups were assessed using one-way analysis of variance (ANOVA). $^{*}P < 0.05$; $^{**}P < 0.01$; $^{***}P < 0.001$; $^{****}P < 0.0001$. NS indicates no difference.

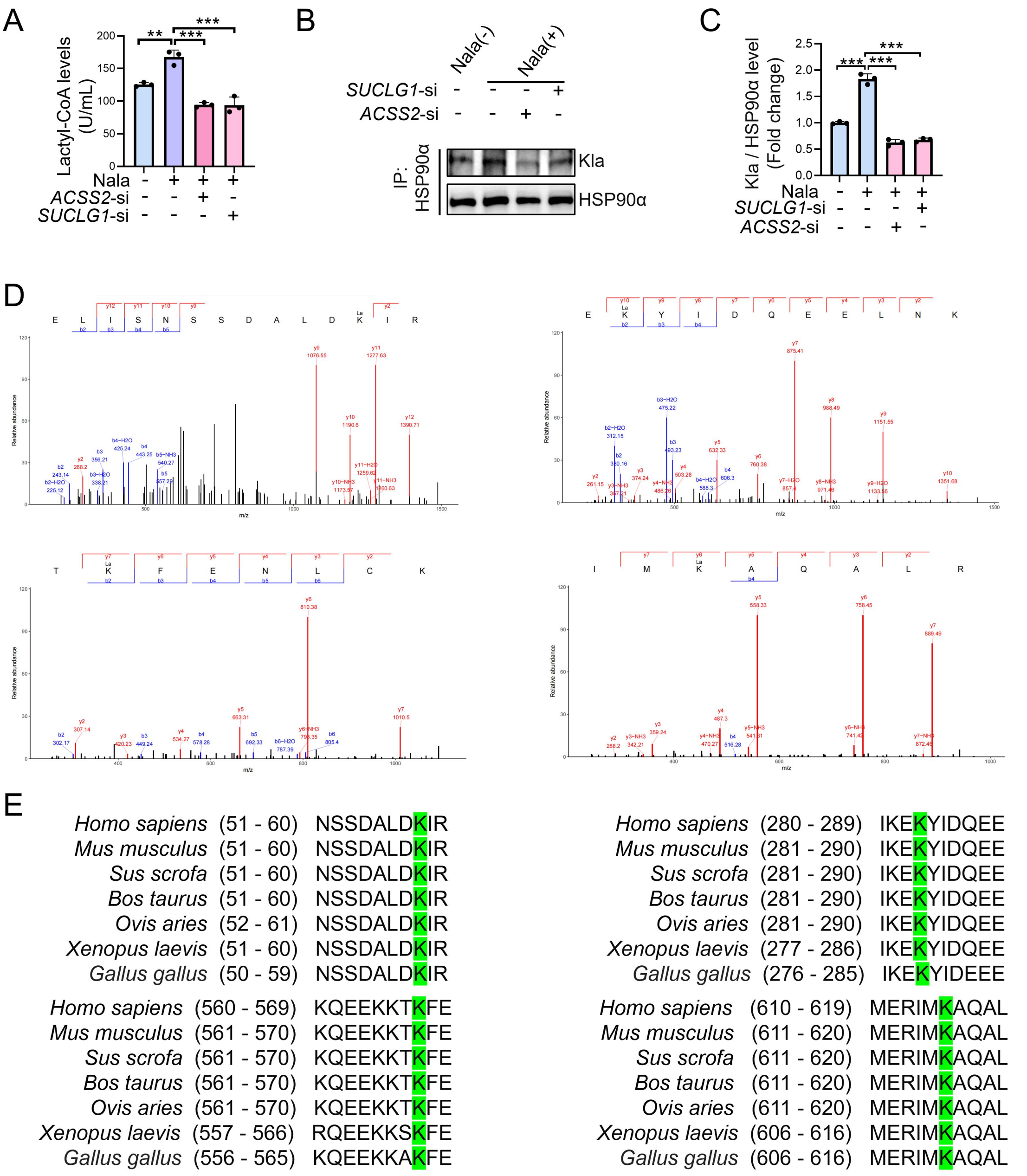

Fig S6. Lactate promotes the lactylation of HSP90α

A Lactyl-CoA levels in KGN cells transfected with *ACSS2* and *SUCLG1* siRNAs followed by sodium lactate treatment. **B** Coimmunoprecipitation analysis of HSP90α lactylation in KGN cells treated as in A. **C** Quantitative analysis of the lactylation level of HSP90α protein shown in B was performed. **D** Mass spectrometry identification of lactylation sites on HSP90α at K58, K284, K568, and K616. Nala: Sodium lactate. The data were presented as mean ± SD. Differences between groups were assessed using one-way analysis of variance (ANOVA). *$P < 0.05$; **$P < 0.01$; ***$P < 0.001$; ****$P < 0.0001$. NS indicates no difference.

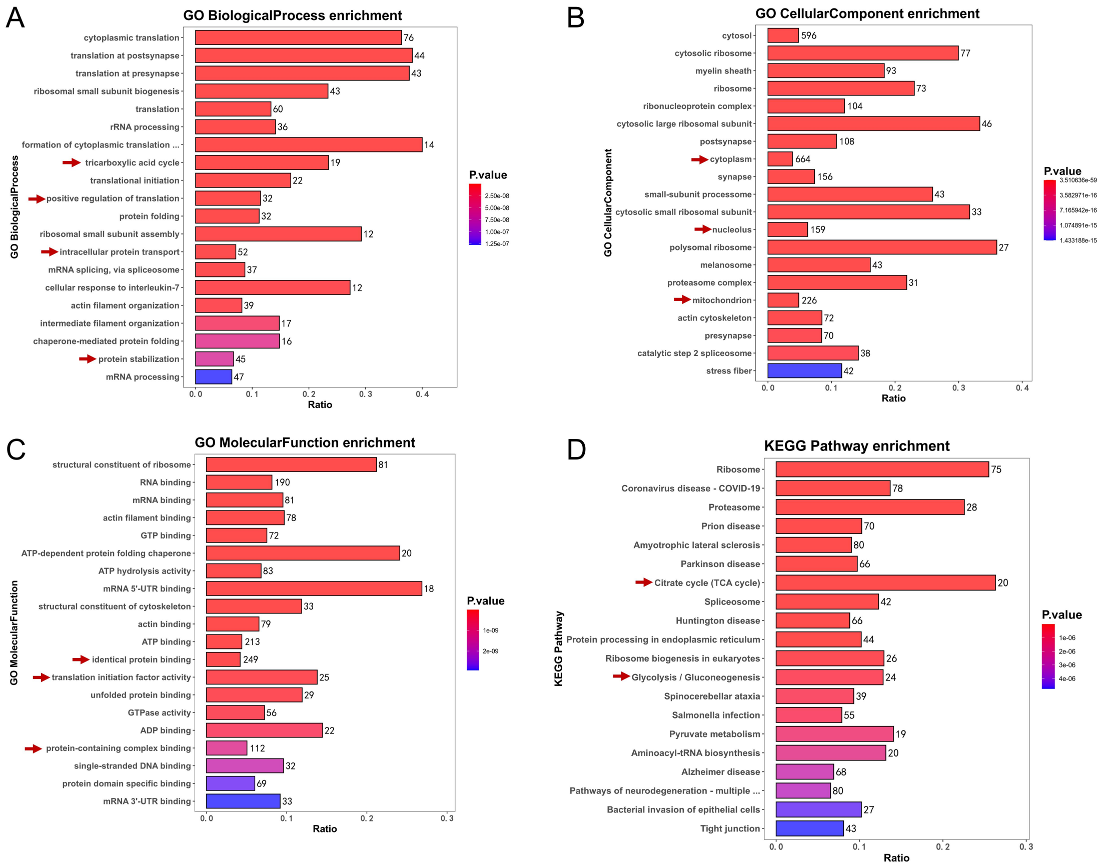

Fig S7. GO and KEGG functional analyses were conducted on HSP90α-interacting proteins

A Gene Ontology (GO) analysis of biological processes for HSP90α-interacting proteins. **B** Gene Ontology (GO) analysis of cellular components for proteins interacting with HSP90 α. **C** Gene Ontology (GO) analysis of molecular functions for HSP90α-interacting proteins. **D** KEGG pathway analysis of the functional pathways associated with HSP90α-binding proteins.

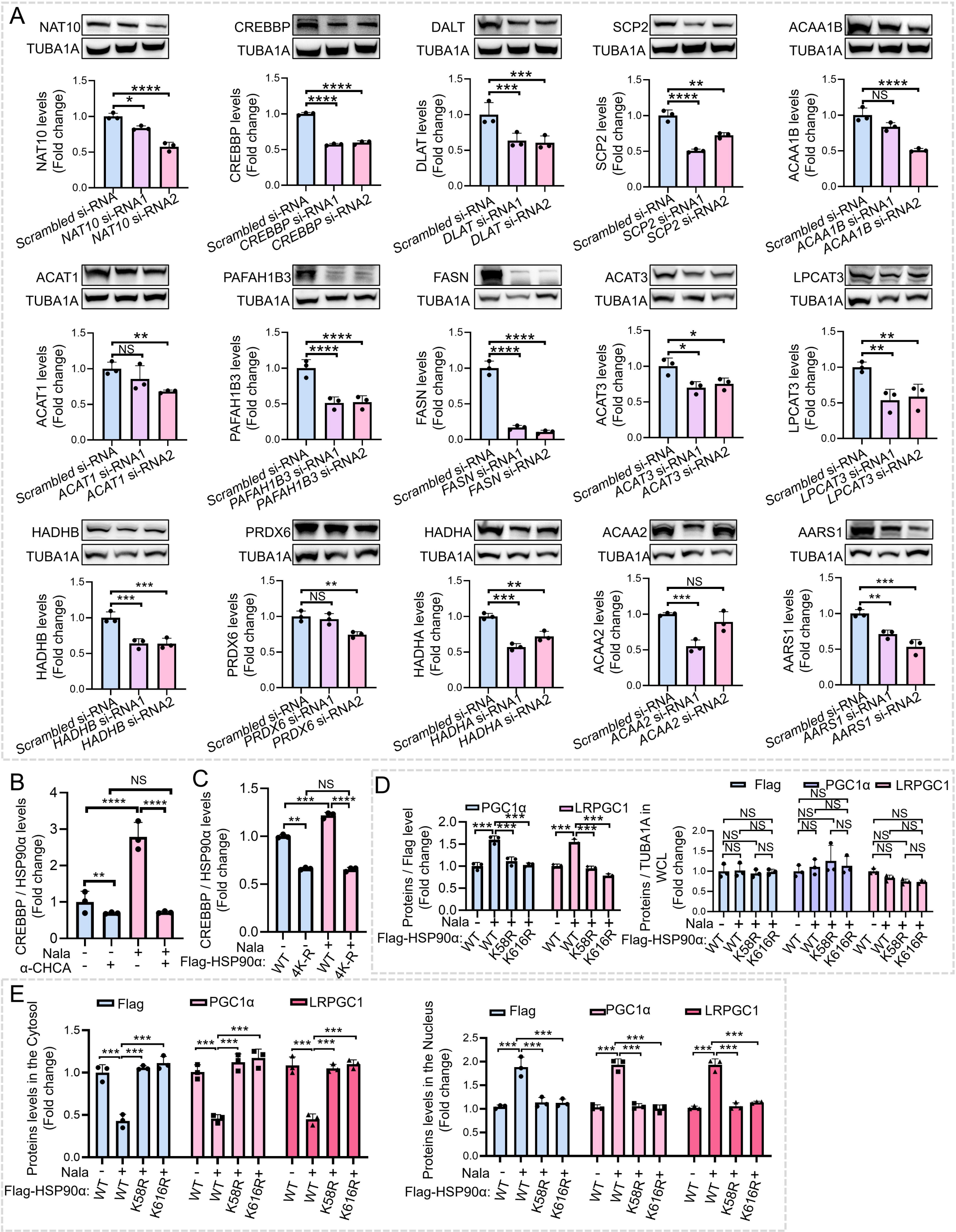

Fig S8. Validation of the knockdown efficiencies of different acyltransferases

A Western blot validation of knockdown efficiency for various acyltransferases in KGN cells. **B** Quantification of CREBBP-HSP90α binding (from Fig. 2G). **C** Quantification of CREBBP-HSP90α binding (from Fig. 2H). **D** Quantification of PGC1α or LRPGC1 coimmunoprecipitated with Flag-HSP90α and corresponding WCL protein levels. (from Fig. 3A). **E** Quantification of Flag-HSP90α, PGC1α, and LRPGC1 levels in

cytoplasmic and nuclear fractions (from Fig. 3F). Nala: sodium lactate. The data were presented as mean ± SD. Differences between groups were assessed using ANOVA. $*P < 0.05$; $**P < 0.01$; $***P < 0.001$; $****P < 0.0001$. NS indicates no difference.

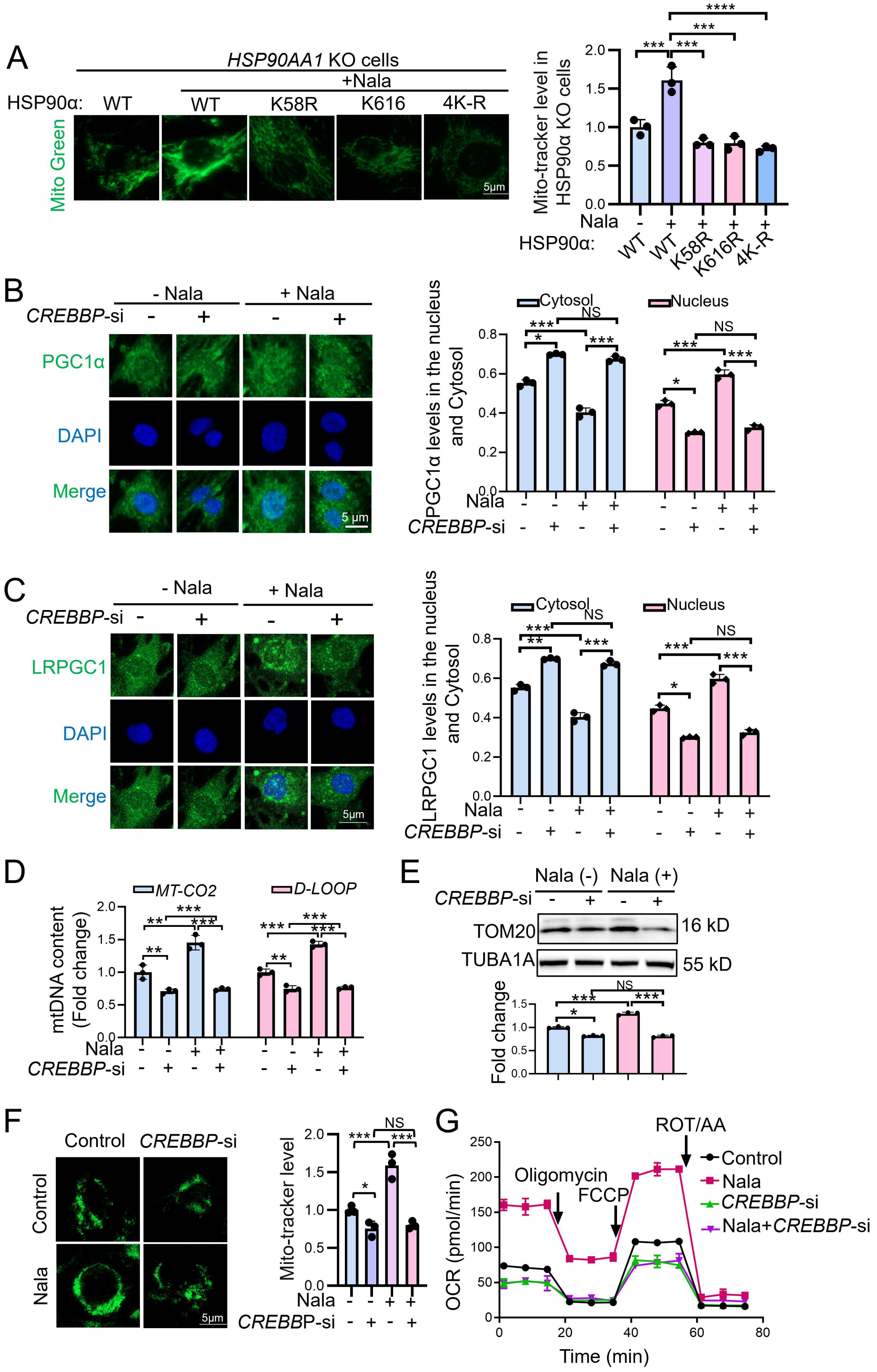

Fig S9. Knockdown of CREBBP suppresses mitochondrial biogenesis

A Mitochondrial content assessed by MitoTracker staining in KGN cells expressing

wild-type or mutant (K58R, K616R, 4K–R) Flag-HSP90α, followed by sodium lactate treatment. Scale bar = 5 µm. **B** Immunofluorescence analysis of PGC1α nucleocytoplasmic translocation in KGN cells transfected with *CREBBP* siRNA and treated with sodium lactate. **C** Immunofluorescence analysis of LRPGC1 nucleocytoplasmic translocation in KGN cells treated as in B. **D** RT-qPCR analysis of mtDNA copy number (*MT-CO2*, *D-loop*) in *CREBBP*-knockdown KGN cells with or without sodium lactate. **E** Western blot analysis of TOM20 protein levels in *CREBBP*-knockdown KGN cells treated with sodium lactate. **F** MitoTracker Green staining for mitochondria in *CREBBP*-knockdown KGN cells treated with sodium lactate. Scale bar = 5 µm. **G** Oxygen consumption rate (OCR) in *CREBBP*-knockdown KGN cells treated with sodium lactate. Nala: sodium lactate. The data were presented as mean ± SD. Differences between groups were assessed using ANOVA. *$P < 0.05$; **$P < 0.01$; ***$P < 0.001$; ****$P < 0.0001$. NS indicates no difference.

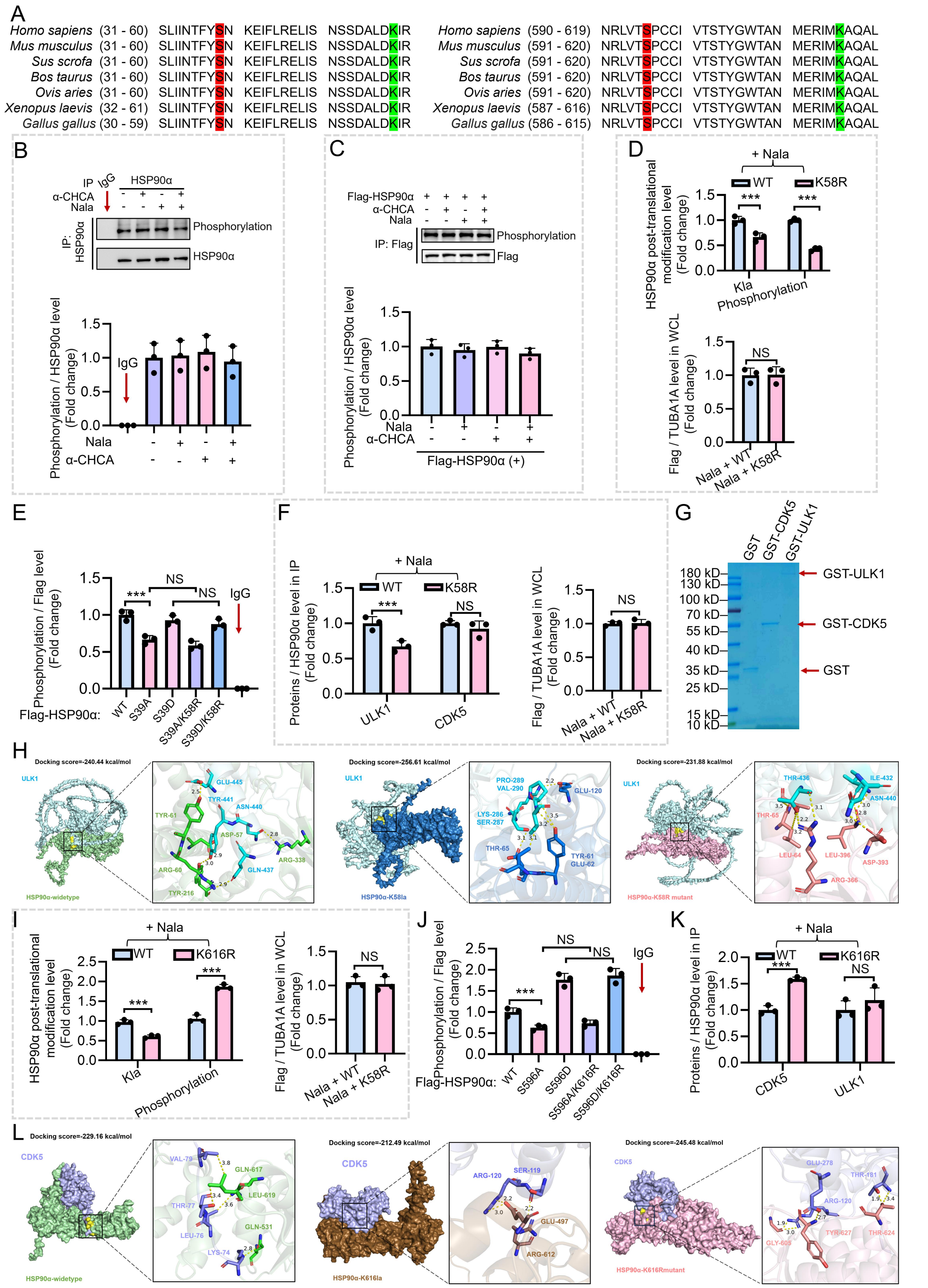

Fig S10. HSP90α lactylation regulates its phosphorylation

A Conservation analysis of HSP90α S39 and S596 phosphorylation sites across species. **B** Coimmunoprecipitation analysis of HSP90α phosphorylation levels in KGN cells pretreated with α-CHCA followed by sodium lactate. **C** Coimmunoprecipitation

analysis of Flag-HSP90α phosphorylation levels in KGN cells transfected with Flag-HSP90α, pretreated with α-CHCA, then sodium lactate. **D** Quantification of HSP90α phosphorylation and lactylation levels (from Fig. 4B). **E** Quantification of HSP90α phosphorylation levels (from Fig. 4C). **F** Quantification of ULK1 and CDK5 binding to HSP90α (from Fig. 4D). **G** SDS-PAGE profile of purified GST-CDK5 and GST-ULK1 proteins. **H** Molecular docking analysis of ULK1 binding to wild-type HSP90α, K58 lactylation-mimetic (K58la), and K58R mutant. **I** Quantification of HSP90α phosphorylation and lactylation levels (from Fig. 4F). **J** Quantification of HSP90α phosphorylation levels (from Fig. 4G). **K** Quantification of ULK1 and CDK5 binding to HSP90α (from Fig. 4H). **L** Molecular docking analysis of CDK5 binding to wild-type HSP90α, K616 lactylation-mimetic (K616la), and K616R mutant. Nala: sodium lactate. The data were presented as mean ± SD. Differences between groups were assessed using ANOVA. *$P < 0.05$; **$P < 0.01$; ***$P < 0.001$; ****$P < 0.0001$. NS indicates no difference.

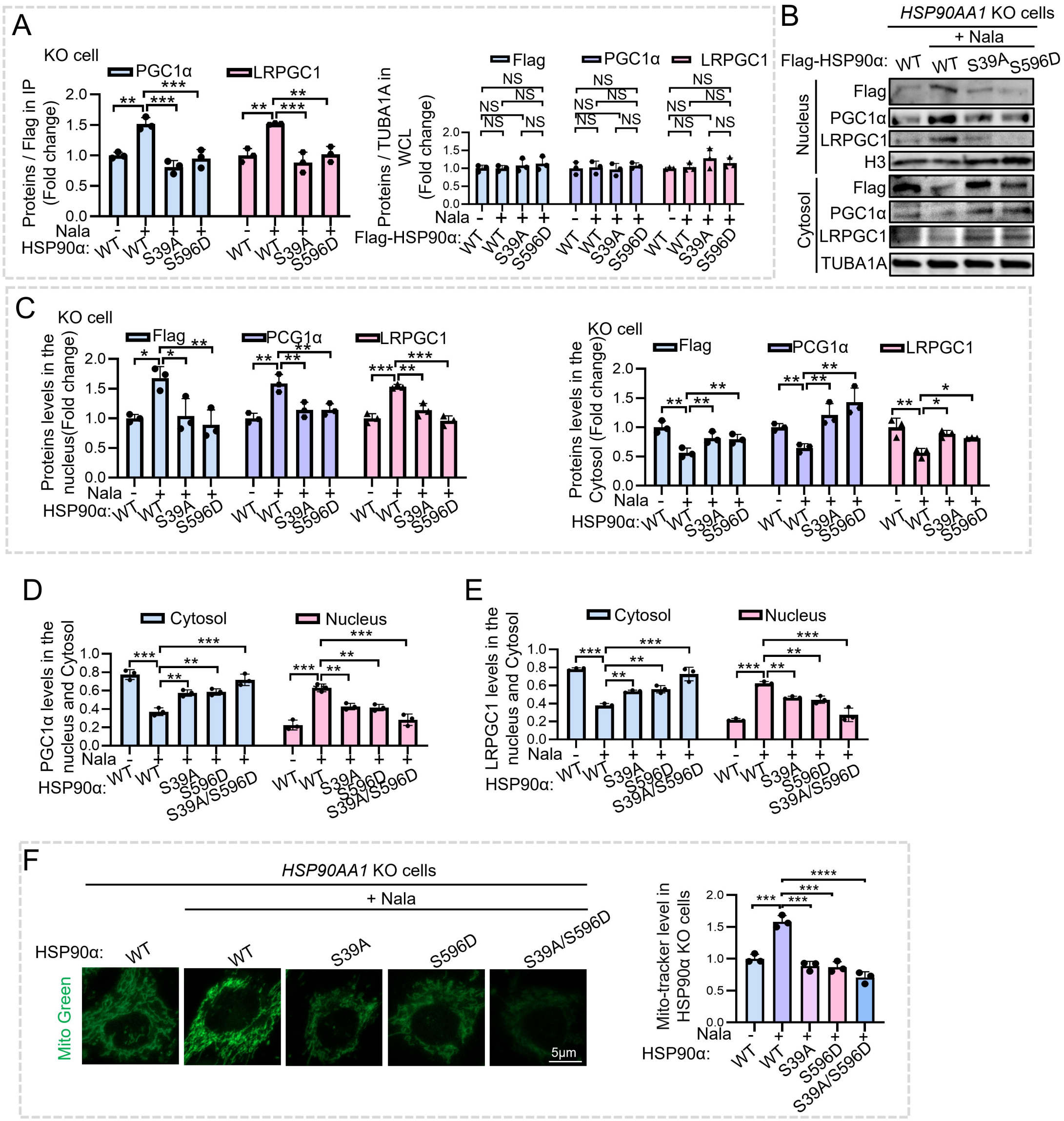

Fig S11. HSP90α S39 and S596 regulate PGC1α/LRPGC1 nuclear translocation and mitochondrial biogenesis.

A Quantification of PGC1α and LRPGC1 binding to HSP90α (from Fig. 4J). **B** Nuclear and cytoplasmic fractionation analysis of PGC1α, LRPGC1, and Flag-HSP90α distribution in *HSP90AA1* KO KGN cells expressing WT, S39A, or S596D, followed by sodium lactate treatment. **C** Quantification of Flag-HSP90α, PGC1α, and LRPGC1 levels in nuclear/cytoplasmic fractions from B. **D** Quantitative analysis of PGC1α fluorescence intensity (from Fig. 4K). **I** Quantitative analysis of LRPGC1 fluorescence intensity (from Fig. 4L). F Mitochondrial content assessed by MitoTracker staining in *HSP90AA1* KO KGN cells expressing WT, S39A, or S596D, followed by sodium

lactate treatment. Scale bar = 5 μm. Nala: sodium lactate. The data were presented as mean ± SD. Differences between groups were assessed using ANOVA. *$P < 0.05$; **$P < 0.01$; ***$P < 0.001$; ****$P < 0.0001$. NS indicates no difference.

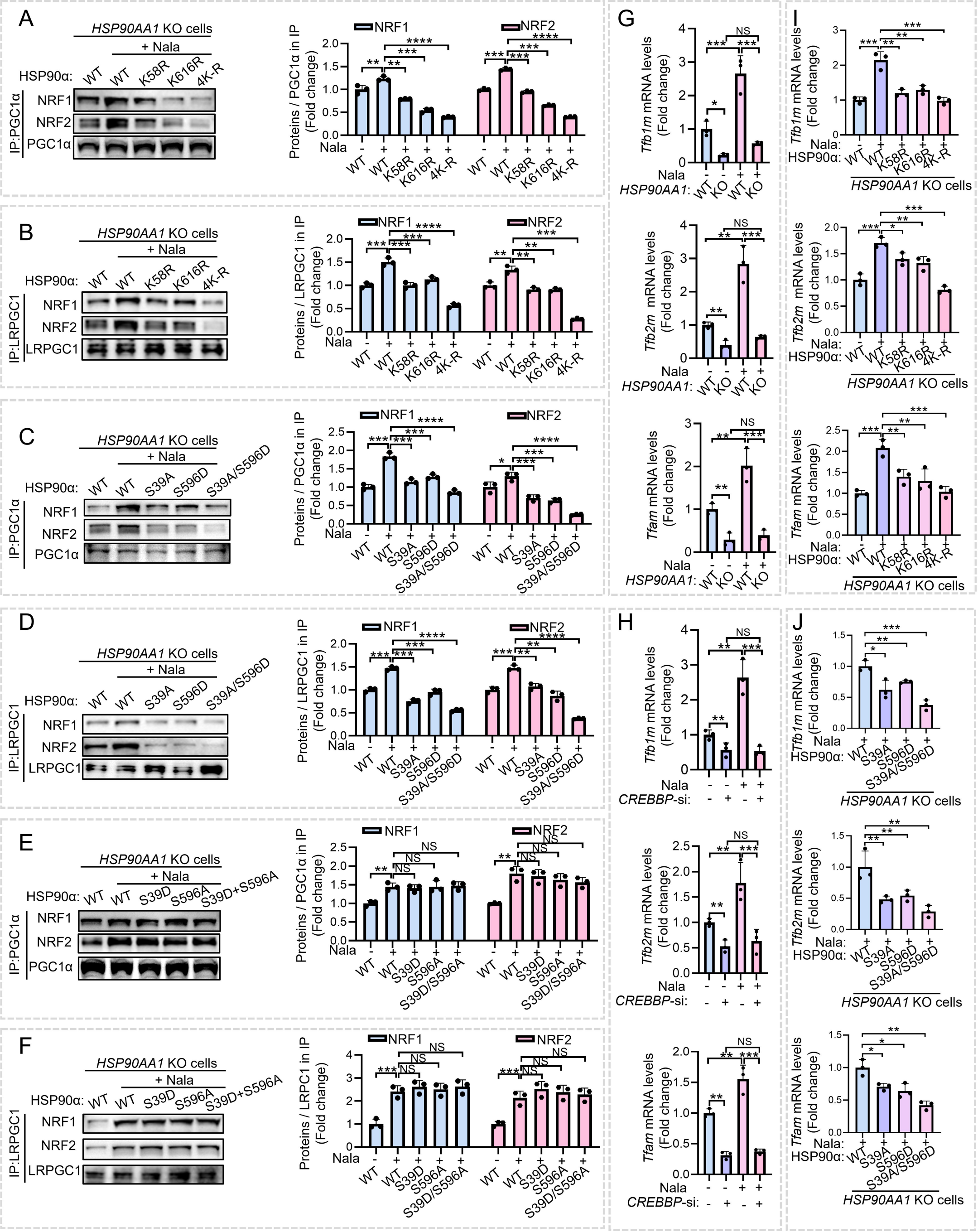

Fig S12. HSP90 lactylation regulates its phosphorylation to promote the binding of PGC1 or LRPGC1 to NRF1/2.

A Coimmunoprecipitation analysis of PGC1α binding to NRF1/NRF2 in sodium lactate-treated *HSP90AA1* KO KGN cells expressing WT, K58R, K616R, or 4K–R. **B** Coimmunoprecipitation analysis of LRPGC1 binding to NRF1/NRF2 in cells treated as in A. **C** Coimmunoprecipitation analysis of PGC1α binding to NRF1/NRF2 in sodium lactate-treated *HSP90AA1* KO KGN cells expressing WT, S39A, S596D, or

S39A/S596D. **D** Coimmunoprecipitation analysis of LRPGC1 binding to NRF1/NRF2 in cells treated as in C. **E** Coimmunoprecipitation analysis of PGC1α binding to NRF1/NRF2 in sodium lactate-treated HSP90AA1 KO KGN cells expressing WT, S39D, S596A, or S39D/S596A. **F** Coimmunoprecipitation analysis of LRPGC1 binding to NRF1/NRF2 in cells treated as in E. **G** RT-qPCR was performed to measure mRNAs expression in WT KGN cells compared to *HSP90AA1* KO KGN cells with treated sodium lactate. **H** RT-qPCR was performed to measure mRNAs expression in *CREBBP* knockdown cells with treated sodium lactate. **I** RT-qPCR analysis of *Tfb1m*, *Tfb2m*, and *Tfam* mRNA levels in sodium lactate-treated *HSP90AA1* KO KGN cells expressing WT, K58R, K616R, or 4K–R. **J** RT-qPCR analysis of *Tfb1m*, *Tfb2m*, and *Tfam* mRNA levels in sodium lactate-treated *HSP90AA1* KO KGN cells expressing WT, S39A, S596D, or S39A/S596D. Nala: sodium lactate. The data were presented as mean ± SD. Differences between groups were assessed using ANOVA. *$P < 0.05$; **$P < 0.01$; ***$P < 0.001$; ****$P < 0.0001$. NS indicates no difference.

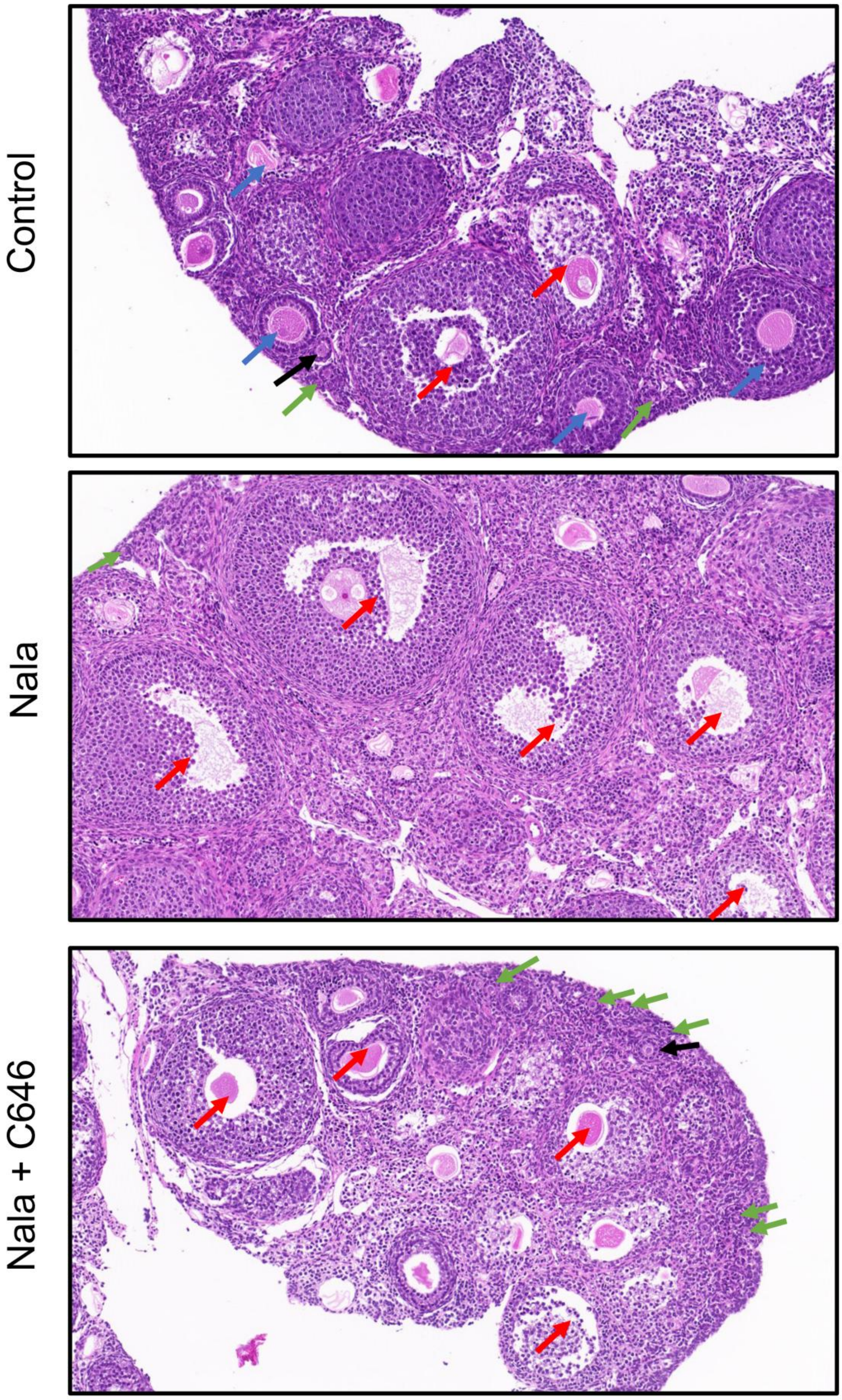

Fig S13. Higher magnification of Fig. 5M.

Green arrows point to primordial follicles, black arrows point to primary follicles, blue arrows point to secondary follicles, and red arrows point to antral follicles.

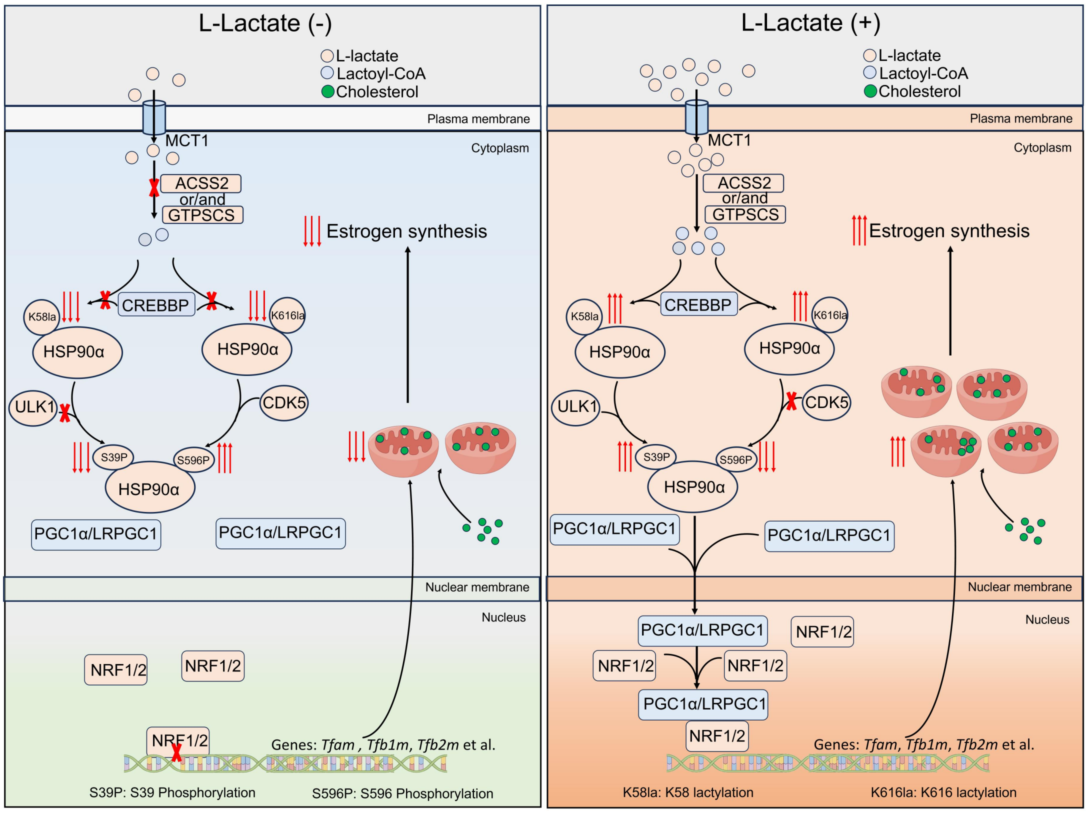

Fig S14. Mechanistic insights into how L-lactate-induced HSP90α lactylation stimulates mitochondrial biogenesis to enhance estrogen production.

The metabolic conversion of L-lactate to Lactyl-CoA is catalyzed by ACSS2 and GTPSCS. Subsequently, CREBBP facilitates site-specific lactylation modifications at K58 and K616 residues of HSP90α. The K58 lactylation serves as a molecular switch that recruits ULK1 kinase to induce S39 phosphorylation, whereas K616 lactylation sterically hinders CDK5-mediated S596 phosphorylation. These coordinated post-translational modifications collectively modulate HSP90α chaperone activity, thereby enhancing nuclear translocation of PGC-1α/LRPGC1 and its functional interaction with nuclear respiratory factors NRF1/2. This cascade ultimately activates transcriptional programs governing mitochondrial biogenesis. The newly generated mitochondria facilitate cholesterol mobilization and its enzymatic conversion to estrogen precursors, establishing a molecular link between cellular metabolic reprogramming and steroid hormone biosynthesis.

Supplementary Table S1. Primer sequences for RT-qPCR

TFAM	forward primer	AGCTCAGAACCCAGATGCAA
	reverse primer	CCACTCCGCCCTATAAGCAT
TFB1M	forward primer	GTTGCCCACGATTCGAGAAA
	reverse primer	TTTCCTTACAATCTTATCTGTCAGC
TFB2M	forward primer	GTTCCTTGGACAGCAGACATC
	reverse primer	TCCGGGATCTGCCATTAGTT

Supplementary Table S2. siRNA sequences

Scrambled siRNA	Sense （5'-3'）	UUCUCCGAACGUGUCACGUTT
	Antisense （5'-3'）	ACGUGACACGUUCGGAGAATT
CREBBP-1979	Sense （5'-3'）	CAGCAAGGCAGAGAUAUUATT
	Antisense （5'-3'）	UAAUAUCUCUGCCUUGCUGTT
NAT10	Sense （5'-3'）	UUGCUGUUCACCCAGAUUAUCTT
	Antisense （5'-3'）	GAUAAUCUGGGUGAACAGCAATT
AARS1	Sense （5'-3'）	CCUCGUGUUCAUCCAGUAUAATT
	Antisense （5'-3'）	UUAUACUGGAUGAACACGAGGTT

DLAT	Sense (5'-3')	GCAGAGGUUGAAACUGAUAAATT
	Antisense (5'-3')	UUUAUCAGUUUCAACCUCUGCTT
SCP2	Sense (5'-3')	GUUGGCUAUGAUAUGAGUAAATT
	Antisense (5'-3')	UUUACUCAUAUCAUAGCCAACTT
ACAA1B	Sense (5'-3')	GAGGGAACCAUGGGAAUAUUUTT
	Antisense (5'-3')	AAAUAUUCCCAUGGUUCCCUCTT
ACAT1	Sense (5'-3')	GUUCGGUCUGGCUAGUAUUUGTT
	Antisense (5'-3')	CAAAUACUAGCCAGACCGAACTT
PAFAH1B3	Sense (5'-3')	GUGCAUUCUGAUGGCACCAUATT
	Antisense (5'-3')	UAUGGUGCCAUCAGAAUGCACTT
FASN	Sense (5'-3')	GCUGGUCGUUUCUCCAUUAAATT
	Antisense (5'-3')	UUUAAUGGAGAAACGACCAGCTT
LPCAT3	Sense (5'-3')	CGAGGAUCUGAGCCUUAACAATT
	Antisense (5'-3')	UUGUUAAGGCUCAGAUCCUCGTT

HADHB	Sense (5'-3')	CCUAUUCGUCAUUCAAGAAAUTT
	Antisense (5'-3')	AUUUCUUGAAUGACGAAUAGGTT
PRDX6	Sense (5'-3')	GGACGCUAACAACAUGCCUGU
	Antisense (5'-3')	AGGCAUGUUGUUAGCGUCCUU
HADHA	Sense (5'-3')	GCUGACCAGAACCCAUAUUAATT
	Antisense (5'-3')	UUAAUAUGGGUUCUGGUCAGCTT
ACAA2	Sense (5'-3')	CACACCUGGUUCAUGAGUUAATT
	Antisense (5'-3')	UUAACUCAUGAACCAGGUGUGTT
PGC1α	Sense (5'-3')	GACGACGAAGCAGACAAGATT
	Antisense (5'-3')	UCUUGUCUGCUUCGUCGUCTT
LRPGC1	Sense (5'-3')	AUUUAUAAAAACAAAUUUGTT
	Antisense (5'-3')	CAAAUUUGUUUUUAUAAAUTT
ACAT3	Antisense (5'-3')	GGCUCCUCACUUGACUCACCU
	Sense (5'-3')	GUGAGUCAAGUGAGGAGCCUU

AARS1	Antisense (5'-3')	CCUCGUGUUCAUCCAGUAUAATT
	Sense (5'-3')	UUAUACUGGAUGAACACGAGGTT

Supplementary Table S3. Primer sequences of Mitochondrial DNA copy number for qRT-PCR.

D-Loop	forward primer	GATTTGGGTACCACCCAAGTATTG
	reverse primer	GTACAATATTCATGGTGGCTGGCA
MT-CO2	forward primer	CCTGCGACTCCTTGACGTTG
	reverse primer	AGCGGTGAAAGTGGTTTGGTT
Beta-actin	forward primer	TCACCCACACTGTGCCCATCTACGA
	reverse primer	CAGCGGAACCGCTCATTGCCAATGG
